# Internet-Delivered Cognitive Behavior Therapy for Adolescents with Obsessive-Compulsive Disorder: An Open Trial

**DOI:** 10.1371/journal.pone.0100773

**Published:** 2014-06-20

**Authors:** Fabian Lenhard, Sarah Vigerland, Erik Andersson, Christian Rück, David Mataix-Cols, Ulrika Thulin, Brjánn Ljótsson, Eva Serlachius

**Affiliations:** Department of Clinical Neuroscience, Centre for Psychiatric Research and Education, Karolinska Institutet, Stockholm, Sweden; University Hospital of Bellvitge-IDIBELL; CIBER Fisiopatología Obesidad y Nutrición (CIBERObn), Instituto Salud Carlos III; Department of Clinical Sciences, School of Medicine, University of Barcelona, Spain

## Abstract

**Background:**

International guidelines recommend Cognitive Behavior Therapy (CBT) as the first line treatment for pediatric obsessive-compulsive disorder (OCD). However, a substantial proportion of patients do not have access to such treatment. We developed and tested the feasibility, efficacy and acceptability of a novel therapist-guided, Internet-delivered CBT (ICBT) platform for adolescents with OCD.

**Methods:**

An interactive, age-appropriate ICBT platform (“BiP OCD”) was developed. Twenty-one adolescents (12–17 years) with a DSM-IV diagnosis of OCD and their parents were enrolled in the study. All participants received 12 weeks of ICBT with therapist support. The primary outcome measure was the Children’s Yale-Brown Obsessive-Compulsive Scale (CY-BOCS). Acceptability was assessed at post-treatment.

**Results:**

Participants completed on average 8.29 (*SD* = 3.0) of the 12 treatment chapters. Treatment yielded significant improvements on all clinician-, parent- and most self-administered outcome measures, with a large effect size of *d* = 2.29 (*95% CI* 1.5–3.07) on the CY-BOCS. Patients continued to improve at follow-up. At 6-month follow-up, 71% were classified as responders (≥35% decrease on the CY-BOCS) and 76% as being in remission (CY-BOCS score ≤12). Average clinician support time was less than 20 minutes per patient per week. The majority of participants felt that BiP OCD was age-appropriate and rated the treatment as good or very good.

**Conclusions:**

ICBT could be efficacious, acceptable, and cost-effective for adolescents with OCD. More rigorously controlled studies are needed to further evaluate the treatment.

**Trial Registration:**

ClinicalTrials.gov; NCT01809990.

## Introduction

Obsessive-compulsive disorder (OCD) is a potentially severe and often chronic mental disorder [1] that affects up to 2% of children and adolescents [2], [3]. Pediatric OCD is commonly associated with severe impairments in academic, social and family functioning [4] and increases the risk of future OCD and other comorbid anxiety, mood, and eating disorders in adulthood [5]–[7]. As duration of illness may predict long-term persistence of the disorder, early intervention is strongly advised [8].

International guidelines recommend cognitive behavior therapy (CBT) as the first-line treatment for pediatric OCD [9], [10]. Several meta-analyses have shown that CBT is an effective treatment for pediatric OCD [11]–[13], with average symptom reductions between 40 and 64% on the Children’s Yale-Brown Obsessive-Compulsive Scale [14]. Despite the existence of effective treatments, many OCD sufferers never seek help and, if they do, they seldom get access to good quality CBT [15], [16]. A lack of suitably trained therapists, psychosocial factors and geographical distances are frequent barriers to accessing such interventions [17], [18].

Internet-delivered CBT (ICBT) is one way of increasing both the accessibility to CBT and treatment capacity. ICBT often includes the same treatment content as regular CBT but entails much less therapist contact. Patients work on their own with web-based treatment material and homework assignments and therapist support is provided through emails and telephone calls. In a recent review of 108 studies, ICBT was found to be an effective and probably cost-effective intervention in the treatment of adults with various psychiatric disorders, with depression, anxiety disorders and chronic pain being the most studied conditions [19]. Furthermore, non-inferiority trials indicate that ICBT is at least as effective as traditional face-to-face CBT for depression, social anxiety disorder and panic disorder [20]–[22]. The efficacy of ICBT for adults with OCD has been demonstrated in two uncontrolled studies and two randomized waitlist-controlled trials, with large within-group as well as between-group effect sizes [23]–[26].

The development of internet-based treatments for children and adolescents with mental disorders lags considerably behind, despite a very high rate of Internet usage in this age group. For example, in Sweden, 90% of young people aged 12–24 years and 96% of school children aged 12–15 years have access to an Internet-connected computer on a daily basis, which makes them the most connected age group in the population [27]. To date, only a handful of case series and open trials [28], [29] and two randomized controlled studies of therapist-supported ICBT for pediatric anxiety disorders [30], [31] have been conducted. To our knowledge, no ICBT protocols exist for pediatric OCD. The aims of this study were to develop and initially test the feasibility, efficacy and acceptability of a novel therapist-guided, ICBT platform for adolescents with OCD.

## Methods

### Participants

The protocol for this trial and supporting TREND checklist are available as supporting information; see **protocol S1 and S2** and **Checklist S1**. The study was approved by the Regional Ethical Review Board in Stockholm, Sweden and registered at clinicaltrials.gov (identifier: NCT01809990). Participants were 21 adolescents with a primary diagnosis of OCD and at least one of their parents. Both parents were invited to participate, though one parent was assigned as the main contact person (76% mothers). The mean time from OCD onset to inclusion in this study was *M* = 3.87 years (*SD* = 2.64). Twenty percent of the participants were on a psychotropic medication and 9 of the 21 had received some form of psychological intervention prior to enrolment in the current study. About two thirds of participants had at least one additional comorbid psychiatric diagnosis besides OCD. **Table 1** gives more detailed information on the demographic and clinical characteristics of the sample.

**Table 1:** Demographic and clinical characteristics of the study sample (*N* = 21).

Variable^a^		*N*	%
**Age (years)**	*M (SD)*	14.4 (2.64)	
	min – max	12.3–17.3	
**Gender**	Girls	13	61.9%
	Boys	8	38.1%
**Adolescent lives with**	Mother	4	19%
	Father	1	4.8%
	Both parents	11	52.4%
	Alternating	5	23.8%
**Ethnicity mothers**	Swedish	17	81%
	Other European	4	19%
**Ethnicity fathers**	Swedish	19	90.5%
	Other European	1	4.8%
	Middle East	1	4.8%
**Ethnicity adolescents**	Swedish	20	95.2%
	Other European	1	4.8%
**Education father**	Primary	3	14.3%
	Secondary	7	33.3%
	University	11	52.4%
**Education mother**	Primary	0	0%
	Secondary	4	19%
	University	17	81%
**Current psychotropic medication**	None	17	81%
	Methylphenidate	3	14.3%
	Antiepileptic	1	4.8%
**Earlier psychological treatments**	None	12	57.1%
	Unspecified	6	28.6%
	CBT (without ERP)	2	9.5%
	CBT (with ERP)	1	4.8%
**Frequency of comorbid diagnoses**	Specific phobia	8	38.1%
	GAD	7	33.3%
	ADHD	5	23.8%
	Separation anx.	3	14.3%
	Social phobia	3	14.3%
	Depression	2	9.5%
	Conduct disorder	1	4.8%
**Number of participants with 0–4 comorbid diagnoses**	None	6	28.6%
	One	5	23.8%
	Two	7	33.3%
	Three	2	9.5%
	Four	1	4.8%
**Duration of OCD**	*M (SD)*	3.87 (2.64)	
	Min – max	0.5–10.4	

*Note* ^a^Abbreviations: CBT = Cognitive Behavior Therapy, ERP = Exposure and response prevention, GAD = Generalized anxiety disorder, ADHD = Attention deficit hyperactivity disorder, Separation anx = Separation anxiety disorder.

Written, informed consent was obtained from all participating adolescents and parents prior to inclusion. Inclusion criteria were: a) a primary DSM-IV-TR diagnosis of OCD, b) a total score of ≥16 on the Children’s Yale-Brown Obsessive-Compulsive Scale (CY-BOCS [14]), c) age between 12 and 17 years, d) ability to read and write Swedish, e) daily access to the Internet, f) a parent that was able to co-participate in the treatment, and g) participants on psychotropic medication must had been on a stable dose for the last 6 weeks prior to baseline assessment. Exclusion criteria were: h) diagnosed autism spectrum disorder, psychosis, bipolar disorder, severe eating disorder, i) suicidal ideation, j) on-going substance dependence, k) not able to read or understand the basics of the ICBT self-help material, l) to have completed a course of CBT for OCD within last 12 months (defined as at least 5 sessions CBT including exposure and response prevention), and m) on-going psychological treatment for OCD or another anxiety disorder.

### Measures

#### Primary outcome measure

Children’s Yale-Brown Obsessive-Compulsive Scale (CY-BOCS, [14]), a semi-structured clinician administered interview for assessment of symptom severity in pediatric OCD.

#### Secondary outcome measures

Children’s Obsessional Compulsive Inventory Revised (ChOCI-R [32]), a self- and parent-report measure of OCD symptom severity. ChOCI-R provides an impairment subscale that is comparable to the CY-BOCS total score (analogous items and scale range between 0–40). Child Obsessive-Compulsive Impact Scale – Revised (COIS-R [33]), a self- and parent-report scale of OCD symptom impact on everyday life. Clinical Global Impression – Severity (CGI- S [34]), a brief clinician rating of symptom severity. Clinical Global Impression - Improvement (CGI-I [35]), a brief clinician rating of the patients’ symptom severity change relative to the baseline assessment. Children’s Global Assessment Scale (CGAS [36]) is an instrument to quantify the overall level of functioning in children and adolescents. Spence Child Anxiety Scale – Child and Parent version (SCAS C/P [37]), a child and parent self-report measure of anxiety. Child Depression Inventory (CDI-S, [38]), a short version of the CDI, measuring levels of depression in young people. Strengths and Difficulties Questionnaire (SDQ [39]), a self- and parent-report measure of general mental health. Family Accommodation Scale, Parent-Report (FAS-PR [40]), a parent-report questionnaire focusing on accommodation behaviors in parents with a child with OCD.

#### Treatment acceptability

At post-treatment, participants answered questions about the acceptability and experience of ICBT, such as “Usually you visit a clinic to meet with a psychologist. In this Internet treatment you had contact with your psychologist via mail and telephone. How did that work for you?” (multiple choice answers, e.g. “I would have preferred to meet my psychologist face-to-face more often.”) and “Which grade do you give this Internet treatment as a whole?” (from “bad” to “very good”).

All self- and parent-rated measures were administered online, a method which has been shown to be reliable and to produce results similar to traditional paper-and-pencil administration [41].

### Procedure

**Figure 1:**
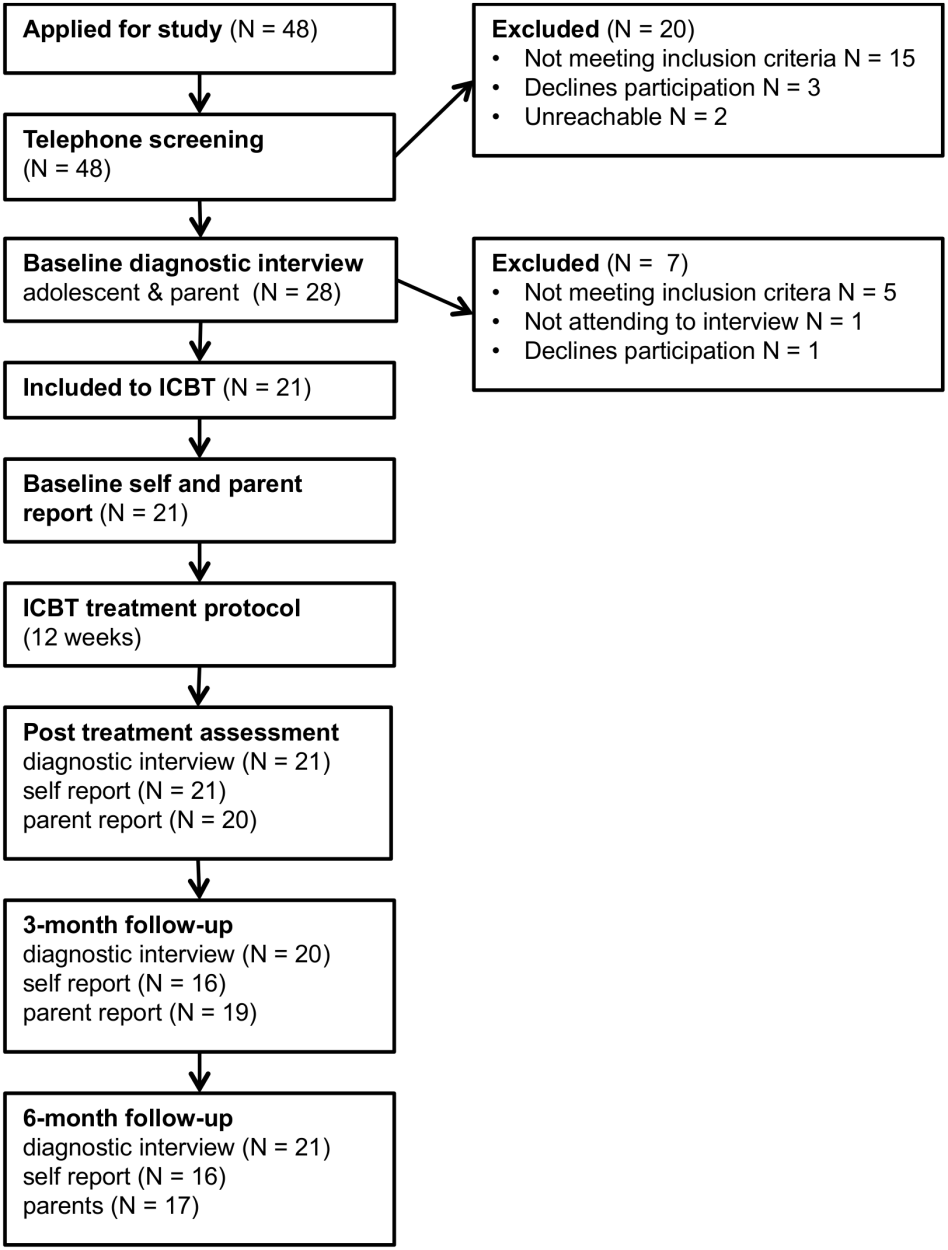
Study flow chart.

Based on power calculations, we aimed to enroll 21 participants in the trial (assuming a large effect size, Coheńs *d* = 0.8, 80% power, *p* = .05). Participants were recruited from January to February 2013 in the Stockholm area through newspaper and Internet advertisements. Information about the study was given on the research group’s homepage at the Child and Adolescent Mental Health Service in Stockholm (www.bup.se/bip). Recruitment was carried out in two steps: telephone screening and face-to-face assessment. **Figure 1** provides an overview of the inclusion, assessment points and treatment procedures.

Applicants registered their interest to participate on the study’s homepage. A clinical psychologist then contacted applicants for an initial telephone interview in order to assess broad inclusion and exclusion criteria. Following this telephone screening, potential participants and their parents were invited to a face-to-face assessments, which included the diagnostic screening interview MINI-KID [42]. Once the diagnosis of OCD was confirmed, the adolescents and their parents were jointly interview with the CY-BOCS. Clinicians administered CGI-I, CGI-S and C-GAS in connection with the interview. Both MINI-KID and CY-BOCS interviews were conducted by experienced clinical psychologists or a final-year clinical psychology student. To assure the reliability of the assessments, pre- and post-treatment CY-BOCS interviews were taped and a random sample (*N* = 19, 45%) of anonymised interviews were rated by two independent evaluators (FL or SV). Inter-class correlations for CY-BOCS inter-rater reliability was *r = .*92, which is excellent according to statistical guidelines [43]. Following face-to-face assessment, participants meeting inclusion criteria were provided with an information sheet and verbal information on the study as well as consent form. Included participants were then asked to fill in self-report measures on the Internet.

At post-treatment, and no later than 4 weeks after the treatment had ended, families were asked to complete all self-administered measures over the Internet and were invited back to our clinic where all clinical interviews were re-administered (CY-BOCS, CGI-I, CGI-S and C-GAS). At 3- and 6-month follow-up, the same measures were administered over the telephone.

### Intervention

**Figure 2:**
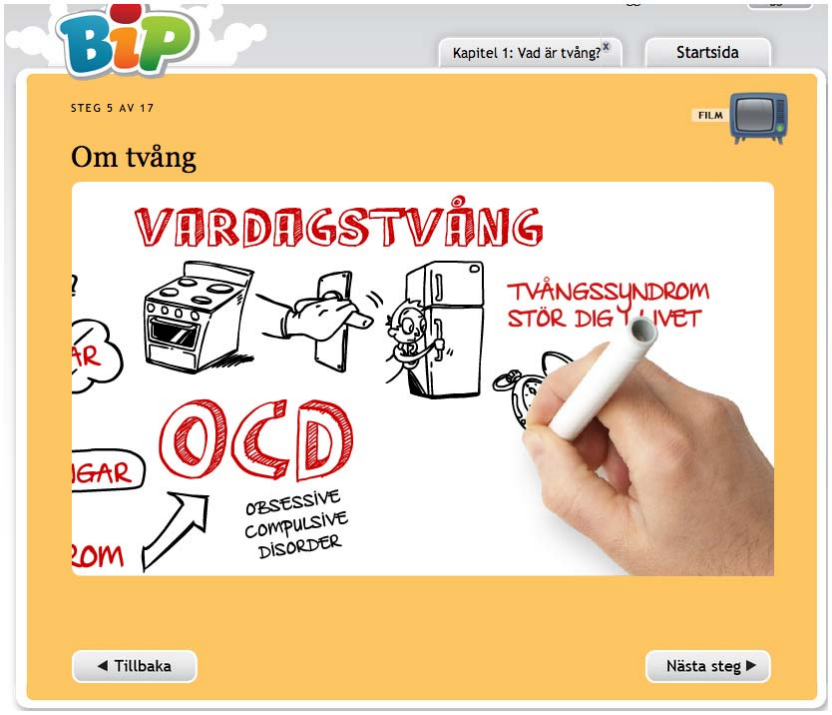
Screenshot of an animated psychoeducation film from BiP OCD (Chapter 1).

The ICBT treatment platform, named “BiP [BarnInternetProjektet] OCD” is completely web-based and designed for use by both adolescents with OCD and their parents. The technical platform is especially designed with age-appropriate appearance, animations and interactive scripts, and was previously tested in an open trial of ICBT for specific phobias in children [29]. The OCD treatment manual was constructed by the research group and consists of evidence-based interventions adapted from other widely researched and validated protocols [44]–[50]. BiP OCD contains 12 chapters designed for the adolescent participants and 5 chapters designed for the parents. The content includes educative texts, films, and exercises. The parent protocol consists of 5 chapters that parallel the adolescent’s part of the treatment and address specific parent-related topics, such as family accommodation and parental coping strategies. BiP OCD is currently available in Swedish and an English version is being prepared. **Table 2** gives an overview of the structure and content of the treatment. Screenshots of some of the relevant sections are provided in **Figure 2** and **Figure S1 and S2**.

**Table 2:** An overview of the content of the ICBT treatment protocol.

Treatment phase	Chapter	Parent chapters	Adolescent chapters
**Psychoeducation**	1	Introduction to ICBT	Introduction to ICBT
	2	About OCD	What is OCD?
	3		We are cracking the code: The OCD circle
	4	Exposure and response prevention	Building a hierarchy
**Exposure with response prevention (ERP)**	5		Testing exposure
	6	Being an exposure coach	Planning your ERP training
	7		New steps with ERP
	8	When the family has OCD	ERP – frequent problems and solutions
	9		More new steps with ERP
	10		Talking back to OCD - Coping with obsessions
**Relapse prevention**	11		The final sprint
	12		Your treatment in the rear-view mirror

During the 12-week treatment period, the therapists had regular contact with participants in order to guide them through the treatment, choose homework assignments and problem-solve when necessary. Participants were instructed to initiate contact with the therapists by sending messages via the ICBT platform whenever they had questions or wanted to discuss any aspects of the treatment. In the case of inactivity of a participant (defined as either no log-ins, no completed assignments or no completed chapters for over one week), the therapist then initiated contact with the family by sending a message via the ICBT platform or directly calling the adolescent or parent. We measured the weekly amount of therapist time spent with each family. Therapist support was given by two licensed clinical psychologists experienced in the treatment of pediatric OCD (FL and SV) and a final year psychology student. Clinicians met once a week for supervision.

### Statistical Analysis

The statistical analyses were planned in advance and carried out with SPSS version 21 (SPSS inc., Chicago) and STATA version 13 (StataCorp Inc.). Hypothesis testing on the primary outcome pre−/post-treatment mean difference was done using two-sided paired t-tests, where no missing data occurred. At 3-month follow-up, there was a single subject with missing CY-BOCS scores; this missing value was replaced by multiple imputation. The imputation model included all longitudinal outcome measures. Ten data sets were generated. Results of identical analyses performed on each of the 10 data sets were combined following Rubin’s rules [51].

Because there were missing data at more than two time points on the secondary outcome variables, linear mixed effects models were implemented for analysis. The models included fixed effects for time (pre-, post-treatment, follow-up), and a random effect for individual subjects. Prior to analysis with mixed effects models the data was checked for the “missing at random” assumption. No significant differences could be found between participants with and without missing data on any baseline characteristics. Moreover, the data was checked for the modeĺs assumption of normally distributed residual errors, showing that our data met this assumption. Ordinal variables were analyzed using Wilcoxon signed ranks test. McNemar test was used for repeated binary variables. Effect sizes on primary and secondary outcomes are reported as Cohen’s *d* = (M_1_– M_2_)/SD_pooled_ with 95% confidence intervals. We defined treatment response as a decrease of ≥35% on the CY-BOCS and remission as a CY-BOCS total score ≤12 [52].

## Results

### Adherence

Adolescents completed on average 8.29 (*SD* = 3.0) of the 12 treatment chapters. Parents completed on average 4.67 (SD = 0.79) of the five parent chapters. Non-completers were defined as adolescents completing fewer than 4 chapters, as chapters 1–4 provide basic psychoeducation and tools for exposure and response prevention. One adolescent did not reach chapter 4, though a closer examination of that case revealed that the participant had understood the rationale and worked successfully with exposure and response prevention despite not logging onto the system. Therefore, we considered that all participants had partaken in the treatment.

### Primary Outcome Results

Means and standard deviations for all outcome measures at four assessments points are presented in **Table 3** and effect sizes in **Table 4**. There was a significant decrease of OCD symptom severity assessed by the CY-BOCS from pre- to post-treatment with *t*(20) = 6.135, *p*<.001 resulting in a large effect size of *d* = 2.29 (*95% CI* 1.5–3.07). The average reduction in CY-BOCS total score from pre to post-treatment was 40.8% (*SD* = 25.35).

**Table 3:** Outcome measures’ means (*M*) and standard deviations (*SD*) at pre-, post-treatment and follow-up^a^.

*Measures* b	pre		post		3-month FU		6-month FU	
*Clinician-rated*	*M*	*SD*	*M*	*SD*	*M*	*SD*	*M*	*SD*
CY-BOCS	21.33	3.54	12.05**	4.51	8.8**	5.11	9.14	6.41
C-GAS	56.1	6.28	71.5**	9.32	73.95	8.99	73.48	9.69
*Self-rated*								
ChOCI-R symptom	13.57	8.71	6.43**	6.6	5.31	6.74	5.00	6.61
ChOCI-R impairment	22.57	8.08	11.62**	6.34	9.88	8.87	10.38	9.09
COIS-R	17.33	15.49	6.57**	7.9	5.19	8.38	6.00	8.99
SCAS OCD subscale	9.05	4.96	4.05**	3.41	2.88	3.76	3.25	3.98
SCAS without OCD	30.43	16.91	20.24**	13.54	18.94	14.01	18.25	14.24
CDI-S	9.62	1.4	9.86	1.15	2.5**	2.73	2.19	2.14
SDQ	13.52	5.52	10.57	4.02	10.69	4.22	10.50	4.84
*Parent-rated*								
*ChOCI-R symptom*	12.35	6.76	6.50**	5.07	5.26	5.58	4.53	4.33
*ChOCI-R impairment*	24.90	7.03	17.8**	9.95	12.37*	8.1	11.47	6.41
COIS-R	25.25	16.09	16.75*	17.15	13.00	15.65	13.88	15.02
FAS-PR	14.60	8.44	9.60*	7.09	6.89	8.05	6.47	6.91
SCAS OCD subscale	7.33	4.22	4.25*	3.24	3.16	3.1	2.76	3.01
SCAS without OCD	25.24	15.73	16.00**	13.54	16.37	12.24	15.65	14.13
SDQ	12.0	6.71	10.3	6.34	10.32	6.63	9.65	6.44

*Note* ^a^Uncorrected means and standard deviations,

b Abbreviations: CY-BOCS = Children Yale-Brown Obsessive Compulsive Scale, C-GAS = Children’s Global Assessment Scale, ChOCI-R symptom/impairment = Children’s Obsessive Compulsive Inventory Revised symptom and impairment scales, COIS-R = Child Obsessive-Compulsive Impairment Scale Revised, SCAS = Spence Child Anxiety Scale, CDI-S = Children’s Depression Inventory – Short version, FAS-PR = Family Accommodation Scale Parent Rating, SDQ = Strength and Difficulties Questionnaire. *p<.05; **p<.001, p values refer to comparisons from pre-treatment to post, post-treatment to 3-month follow-up, and 3-month follow-up to 6-month follow-up, respectively.

**Table 4:** Outcome measures’ effect sizes, *ES* (Cohen’s *d*) at pre-, post-treatment and follow-up.

*Measures* ^a^	pre/post		post/3-month FU		3-month FU/6-month FU		pre/6-month FU	
*Clinician-rated*	*ES*	*(95% CI)*	*ES*	*(95% CI)*	*ES*	*(95% CI)*	*ES*	*(95% CI)*
CY-BOCS	2.29	(1.50–3.07)	0.67	(0.04–1.3)	0.05	(−0.56–0.66)	2.35	(1.50–3.18)
C-GAS	−1.94	(−2.60–−1.12)	−0.27	(−0.93–0.30)	−0.06	(−0.67–0.55)	−2.12	(−2.90–−1.32)
*Self-rated*								
ChOCI-R symptom	0.92	(0.28–1.56)	0.17	(−0.49–0.82)	0.05	(−0.65–0.74)	1.09	(0.38–1.78)
ChOCI-R impairment	1.51	(0.81–2.18)	0.23	(−0.42–0.88)	−0.06	(−0.75–0.64)	1.43	(0.68–2.16)
COIS-R	0.88	(0.24–1.50)	0.17	(−0.48–0.82)	−0.09	(−0.79–0.60)	0.86	(0.18–1.54)
SCAS OCD subscale	1.17	(0.51–1.83)	0.33	(−0.33–0.98)	−0.10	(−0.79–0.60)	1.27	(.55–1.98)
SCAS without OCD	0.67	(0.04–1.28)	0.09	(−0.56–0.74)	0.05	(−0.64–0.74)	0.77	(0.09–1.44)
CDI-S	−0.19	(−0.79–0.42)	3.7	(2.61–4.77)	0.13	(−0.57–0.82)	4.24	(2.88–5.58)
SDQ	0.61	(−0.01–1.23)	−0.03	(−0.68–0.62)	0.04	(−0.65–0.73)	0.58	(0.09–1.24)
*Parent-rated*								
*ChOCI-R symptom*	0.94	(0.29–1.58)	0.23	(−0.4–0.86)	0.15	(−0.51–0.80)	1.31	(0.59–2.01)
*ChOCI-R impairment*	0.79	(0.15–1.42)	0.60	(−0.05–1.24)	0.12	(−0.53–0.78)	1.94	(1.15–2.71)
COIS-R	0.45	(−0.17–1.07)	0.23	(−0.4–0.86)	−0.06	(−0.71–0.60)	0.67	(0.00–1.32)
FAS-PR	0.60	(−0.03–1.22)	0.36	(−0.28–0.99)	0.06	(−0.60–0.71)	1.00	(0.31–1.67)
SCAS OCD subscale	0.82	(0.17–1.45)	0.34	(−0.29–0.97)	0.13	(−0.53–0.78)	1.22	(0.52–1.92)
SCAS without OCD	0.63	(0.0–1.25)	−0.03	(−0.66–0.59)	0.05	(−0.60–0.71)	0.64	(−0.02–1.29)
SDQ	0.29	(−0.33–0.91)	0.0	(−0.63–0.63)	0.10	(−0.55–0.76)	0.39	(−0.26–1.03)

*Note* ^a^Abbreviations: CY-BOCS = Children Yale-Brown Obsessive Compulsive Scale, C-GAS = Children’s Global Assessment Scale, ChOCI-R symptom/impairment = Children’s Obsessive Compulsive Inventory Revised symptom and impairment scales, COIS-R = Child Obsessive-Compulsive Impairment Scale Revised, SCAS = Spence Child Anxiety Scale, CDI-S = Children’s Depression Inventory – Short version, FAS-PR = Family Accommodation Scale Parent Rating, SDQ = Strength and Difficulties Questionnaire.

Twelve participants (57%, *95% CI* 37–76%) responded to the treatment (symptom decrease of ≥35% on the CY-BOCS). Ten participants (48%, *95% CI* 28–68%) had a CY-BOCS score ≤12 at post-treatment and were classed as being in remission.

### Secondary Outcome Results

The mean difference in clinician-rated symptom severity (CGI-S) from pre- to post-treatment was 1.8 points (*SD* = 1.50), representing a statistically significant improvement (*Z = *−3.412, *p*<.01). Eleven participants (52%, *95% CI* 32–72%) were rated as “much improved” or “very much improved” on the CGI-I. All self- and parent-rated measures of OCD symptom severity and impairment as well as family accommodation improved significantly with moderate to large effect sizes (*d* = 0.45–1.51). No significant improvements were observed on CDI-S (depression) and SDQ (general mental health).

### Follow-up Results

There was a further significant decrease on the CY-BOCS from post-treatment to 3-month follow-up (*Z* = −3.63; *p*<.001) with a moderate effect size indicating that patients continued to improve over this time period. At 6 months no further significant changes on CY-BOCS occurred. 71% (*95% CI* 50–86%) of participants were classed as responders both at 3 and 6 months (defined as decrease of ≥35% on the CY-BOCS). The average symptom reduction on the CY-BOCS total score from pre-treatment to 3-months follow-up was 56.6% (*SD* = 26.52) and from pre-treatment to 6- months follow-up 55.0% (*SD* = 32.23). At the 3-month follow-up 62% (*95% CI* 41–79%) of the patients were in remission (CY-BOCS score of ≤12), whereas 76% (*95% CI* 55–89%) of the patients were in remission at 6-month follow-up.

**Figure 3:**
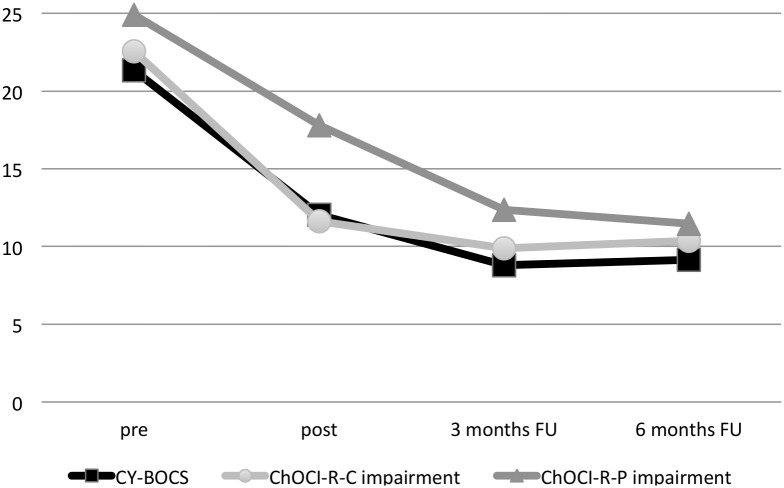
Mean CY-BOCS and adolescent- respective parent-rated ChOCI-R impairment subscale scores at pre-treatment, post-treatment and follow-up.

There were also significant severity changes from post-treatment to 3-month follow-up on the clinician-rated CGI-S (*Z* =  −2.653, *p* = .008) and a non-significant trend for the CGI-I (*Z* = −1.941, *p = *.052). CGI-S and CGI-I scores did not change significantly from 3- to 6-month follow-up. The mean difference from pre-treatment to 6-month follow-up on the CGI-S was 2.35 points (*SD = *1.37). At 3 and 6 months, 71% (*95% CI* 50–86%) of participants were rated as “much improved” or “very much improved” on the CGI-I. No further significant changes from post-treatment to follow-up occurred on self- or parent-rated secondary outcome measures, except for a decrease in parent-rated ChOCI-R impairment and a decrease in self-rated CDI-S at 3 months (see **Table 3** and **4** for details). **Figure 3** displays means on clinician rated CY-BOCS scores and the corresponding self- and parent-rated ChOCI-R impairment subscales.

### Amount of Clinician Support

The average total clinician time per participant (including e-mails and telephone calls to both adolescents and parents) was 233.8 minutes (*SD* = 153.7), which equals to 19.5 minutes per week and participant.

### Treatment Acceptability

Overall, all 21 participants rated the treatment as “good” or “very good”. Nineteen of 21 participants found the treatment age-appropriate. Seventeen of 21 felt comfortable with working only via Internet without meeting the clinician face-to-face, whereas 4 participants would have preferred occasional face-to-face contact with the clinician, in addition to ICBT. An individual analysis of these 4 cases did not indicate any difference in treatment outcome with 3 of the 4 being responders.

## Discussion

We tested the efficacy and acceptability of a novel Internet-delivered CBT (ICBT) platform for adolescents with OCD and their primary caregivers. BiP OCD uniquely combines available evidence-based techniques with age-appropriate interactive materials, films, and animations, making it particularly suitable for this age group.

The participants experienced a significant decrease of OCD symptom severity as well as in OCD-related impairment at post-treatment. Moreover, the treatment had beneficial effects on comorbid anxiety levels and general functioning. In addition, we observed a delayed effect on comorbid levels of depression at 3-month follow-up, a finding that could be interpreted as a secondary effect of reduced OCD symptom impact on everyday functioning. Clinician-rated, self-rated as well as parent-rated measures coherently showed moderate to large within group effect sizes. Furthermore, treatment effects on OCD severity and other secondary measures continued throughout the follow-up period, indicating the sustained benefits of the intervention. The program was consistently rated as age-appropriate and all participating families rated the treatment as being good or very good overall. The majority of participants also felt comfortable working on the Internet with brief telephone and online therapist support.

Interestingly, the correspondence between clinician (CY-BOCS) and adolescent self-ratings (ChOCI-R) of OCD severity in our study was better than the correspondence between adolescent self-ratings and parent ratings. Similarly, we observed a discrepancy between the adolescent self-ratings and parent ratings of OCD impact on everyday life functioning (COIS-R). In both cases, parents provided higher ratings, indicating higher symptom severity and poorer everyday life functioning, though the clinician, self- and parent ratings tended to converge at the 6-month follow-up. In our experience, parents sometimes have limited insight on the occurrence and impact of some OCD symptoms, particularly obsessions of potentially embarrassing content.

### Limitations

This open trial is limited by the absence of a control condition. Consequently, it was not possible to effectively blind participants or assessors and to control for non-specific factors such as expectancy, effects of assessments or clinician contact. Yet, prior research shows that independent evaluators and non-blinded treating clinicians have a high level of agreement in global ratings of OCD symptom improvement [53]. Moreover, considering the high rates of chronicity in OCD [54], [55], the response and remission rates observed in this study are unlikely to be caused by spontaneous remission. Nevertheless, the results should be considered preliminary until a controlled trial is conducted with blind assessments and, ideally, with a credible control condition. Second, by recruiting participants via media and newspapers, we may have introduced important selection biases; the high rates of university degrees in the parents indicate that the sample represents a selected socio-economic group. Thus, although the participants had substantial levels of OCD symptom severity, impairment and comorbid diagnoses, this may have been a particularly motivated group and therefore the generalizability of the results to routine clinical settings remains to be established. Third, the sample consisted mainly of moderately severe cases of OCD and therefore the findings may not generalize to more severe or complex cases. Finally, a longer follow-up of the patients would be helpful to establish the durability of the treatment outcomes reported here.

### Future Directions

The magnitude of ICBT treatment effect on the primary outcome measure (CY-BOCS) was comparable to that of previous studies of standard face-to-face CBT for OCD [44]–[48]. Crucially, the average therapist time spent on supporting patients through ICBT (on average 19.5 minutes per week and participant) was approximately one third of the time usually spent in standard CBT. Therefore, ICBT could potentially be a cost-effective intervention for adolescents with OCD, as it has been shown in many adult ICBT studies in other behavioral and psychiatric problems [19]. An important research question for the future will be how much therapist support is required to achieve optimal cost-effectiveness without impairing efficacy. The literature suggests that the total absence of therapist support is likely to lead to high drop out rates and poorer treatment outcomes [56]. Moreover, it has been shown that proactively scheduled therapist phone support is associated with enhanced treatment adherence and better outcomes, when compared to patient-requested telephone calls in computer-aided self-help for adults with OCD [57]. Future trials manipulating the amount of clinician support could provide valuable information in this regard. Other central questions for the future are the identification of the patient groups that are more likely to benefit from ICBT and how this treatment modality could be used to complement and streamline regular clinical care for OCD.

Possible further development of ICBT may involve the combination of web-based ICBT with integrated smartphone applications. Many everyday activities, such as Internet banking, social media or training applications, are currently both web- and smartphone-based. Smartphone applications have the potential to enhance certain aspects of web-based ICBT. For example, such an application could prompt the patient to engage in exposure and response prevention homework between sessions, thus potentially increasing adherence and overall outcomes [58]. A small number of studies have evaluated smartphone as the solitary format of treatment delivery [59], underlining the potential of smartphone applications as an extension of ICBT. Another promising add-on to ICBT would be videoteleconferencing and web-camera approaches, as a way to deliver face-to-face CBT to patients living in remote areas [60], [61].

Finally, it will be important to study how to best utilize internet-delivered interventions in relation to face-to-face interventions. Considering the potential cost and time savings associated with ICBT, such interventions could perhaps be employed in a stepped-care manner, with ICBT as a first-line intervention and face-to-face CBT given to patients with more severe OCD or higher complexity [62], [63]. Intuitively, this could lead to a better distribution of scarce health care resources, with better access to standardized evidence-based treatment in primary and secondary care settings, and freeing more therapist resources for those in need of more highly specialized care. A different healthcare model may be to employ ICBT as an add-on or complement to standard CBT [64]. Future studies manipulating the sequence of ICBT and face-to-face CBT and incorporating a health-economic component are needed to evaluate these important questions.

### Conclusion

BiP OCD is a therapist-guided, internet-delivered CBT program designed for use by both adolescents with OCD and their parents or primary caregivers. ICBT requires only a fraction of therapist support, compared to standard face-to-face CBT. If the encouraging results of this open trial were replicated in more rigorously controlled studies, in relation to active control conditions and in routine clinical settings, ICBT could greatly increase the availability of effective psychological treatments for adolescents with OCD.

## Supporting Information

Figure S1 **Screenshot from BiP OCD - The OCD cycle (Chapter 2).**
(TIFF)

Figure S2 **Screenshot from BiP OCD - Exercise on CBT and patient-therapist conversation (Chapter 4).**
(TIFF)

Checklist S1 **TREND checklist.**
(PDF)

Protocol S1 **Trial Protocol (Swedish).**
(PDF)

Protocol S2 **Trial Protocol (English).**
(PDF)
