# Supplementary figures and images for: Internet-Delivered Cognitive Behavior Therapy for Adolescents with Obsessive-Compulsive Disorder: An Open Trial
Source: PLoS One. 2014 Jun 20;9(6):e100773. doi: 10.1371/journal.pone.0100773 (PMC4065049; doi:10.1371/journal.pone.0100773)

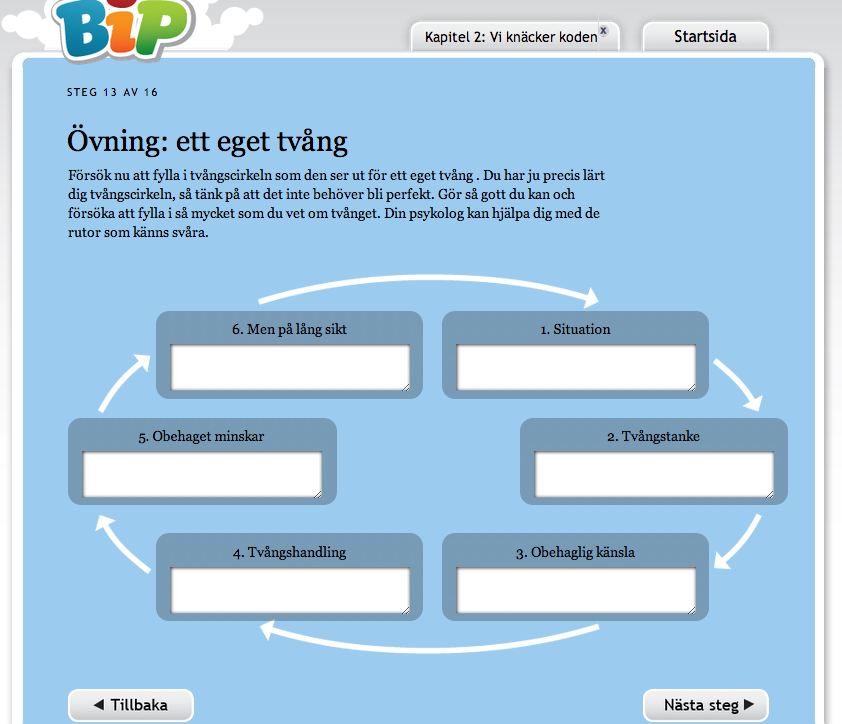

Supplement: Figure S1 — Screenshot from BiP OCD - The OCD cycle (Chapter 2). (TIFF) [file pone.0100773.s001.tiff]

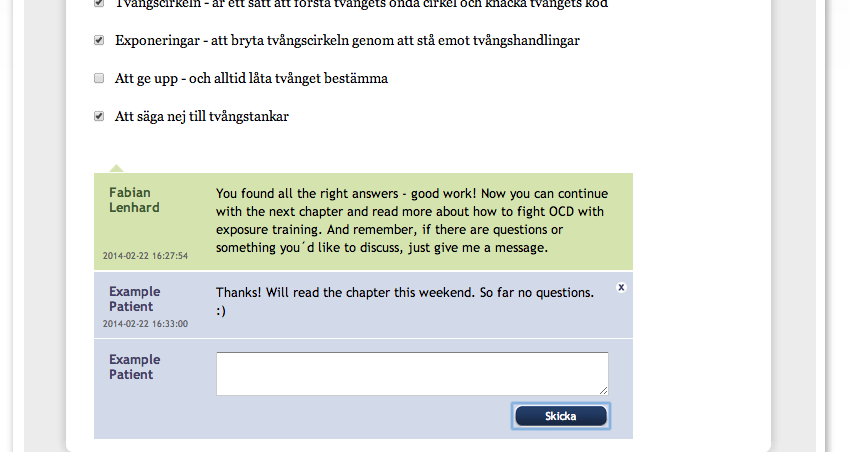

Supplement: Figure S2 — Screenshot from BiP OCD - Exercise on CBT and patient-therapist conversation (Chapter 4). (TIFF) [file pone.0100773.s002.tiff]
