# Supplementary material for: Internet-Delivered Cognitive Behavior Therapy for Adolescents with Obsessive-Compulsive Disorder: An Open Trial
Source: PLoS One. 2014 Jun 20;9(6):e100773. doi: 10.1371/journal.pone.0100773 (PMC4065049; doi:10.1371/journal.pone.0100773)
Supplement: Protocol S1 — Trial Protocol (Swedish). (PDF) [file pone.0100773.s004.pdf]

Beslutad 2012-03-20

ANSÖKAN OM ETIKPRÖVNING AV FORSKNING SOM AVSER MÄNNISKOR

Information till ansökan, ***se Vägledning till ansökan (www.epn.se)***
Beroende på vilken forskning som ansökan gäller kommer de uppgifter som efterfrågas att ha olika relevans. Vid ändring av tidigare godkänd ansökan, se *Vägledning till ansökan*.

Till Regionala etikprövningsnämnden i: Stockholm

Den regionala etikprövningsnämnd till vars upptagningsområde forskningshuvudmannen hör, se respektive nämnd *(www.epn.se).*

Avgift inbetald datum:

Observera att en ansökan aldrig är komplett och därmed kan behandlas förrän blanketten är korrekt ifylld och avgiften är betald.

Projekttitel: Internet-levererad kognitiv beteendeterapi för ungdomar med tvångssyndrom - en pilotstudie

Ange en beskrivande titel på svenska för lekmän. Titeln ska ej innehålla sekretesskyddad information. Ange också i förekommande fall, t.ex. vid klinisk läkemedelsprövning, projektets identitet, forskningsplanens/protokollets nummer, version, datum. Vid ändring av tidigare godkänd ansökan, se Vägledning till ansökan.

Projektnummer/identitet: Version nummer: EudraCT nr (vid läkemedelsprövning):

Uppgifter som fylls i av den regionala etikprövningsnämnden

Ansökan komplett:	Dnr:
Begäran om ytterligare information (i sak):	Begärd information inkommen:
Beslutsdatum:	Expeditionsdatum:

Ansökan avser (gäller även vid begäran om rådgivande yttrande):

Forskning där endast en forskningshuvudman deltar (5 000 kr)	☒
Forskning där mer än en huvudman deltar (16 000 kr)	☐
Forskning där mer än en forskningshuvudman deltar, men där samtliga forskningspersoner eller forskningsobjekt har ett omedelbart samband med endast en av forskningshuvudmännen (5 000 kr)	☐

Endast behandling av personuppgifter (5 000 kr) ☐
(När enbart redan befintliga personregister ska användas, t. ex. nationella databaser)

Forskning som gäller klinisk läkemedelsprövning (16 000 kr) ☐

Ändring av tidigare godkänd ansökan enligt 4 § förordning (2003:615) om etikprövning av forskning som avser människor (2 000 kr) ☐

Om nämnden finner att forskningsprojektet inte faller inom etikprövninglagens tillämpningsområde önskas ett rådgivande yttrande. (Se 4a och 4b §§ i förordning 2003:615 och Vägledning till ansökan)
Ja: ☒ Nej: ☐

1. Information om forskningshuvudman m.m.

1:1 Forskningshuvudman (Se p. 1:1 i Vägledning till ansökan)

Ansökan om etikprövning av forskning ska göras av forskningshuvudmannen. *Med forskningshuvudman avses en statlig myndighet eller en fysisk eller juridisk person i vars verksamhet forskningen utförs.*

Namn: Barn och ungdomspsykiatrin, Stockholms Läns Sjukvårdsområde

Adress:

BUP-divisionen
Barn och ungdomspsykiatri
Box 17564
118 91 Stockholm

1:2 Behörig företrädare för forskningshuvudmannen

Behörig företrädare är t.ex. prefekt, enhetschef, verksamhetschef. Forskningshuvudmännen bestämmer själva, genom interna arbets- och delegationsordningar eller genom fullmakt, vem som är behörig att företräda forskningshuvudmannen.

Namn: Olav Bengtsson Tjänstetitel: Divisionschef

Adress:

BUP-divisionen
Barn och ungdomspsykiatri
Box 17564
118 91 Stockholm

1:3 Forskare som är huvudansvarig för genomförandet av projektet (kontaktperson)

(Se p. 9 bil. nr 10 och p. 1:3 i Vägledning till ansökan)

Observera! Den som är huvudansvarig forskare ansvarar för att andra medverkande som ska genomföra projektet har tillräcklig kompetens (vetenskaplig och klinisk) och vid läkemedelsprövning har tillräcklig kunskap om ”Good Clinical Practice” (GCP). Vid doktorandstudier är som regel handledaren huvudansvarig forskare.

Namn: Eva Serlachius Tjänstetitel: Överläkare, Med Dr

Postadress: BUP-divisionen

Barn och ungdomspsykiatri

Box 17564

118 91 Stockholm

E-postadress: eva.serlachius@ki.se

Telefon:

Mobiltelefon: 0707375225

1:4 Andra medverkande (Se p. 9 bil. nr 1 och p. 1:4 i Vägledning till ansökan)

Övriga deltagande forskningshuvudmän samt forskare ansvariga för att lokalt genomföra projektet (kontaktpersoner) anges här eller i bilaga med namn och adresser (se p. 9 bil. nr 1).

se bilaga nr. 1

1:5 Redovisa tillgång till nödvändiga resurser under projektets genomförande

(Se p. 9 bil. nr 9 och p. 1:5 i Vägledning till ansökan)

Ange vem/vilka som har ansvaret (prefekt, verksamhetschef eller motsvarande) för forskningspersonernas säkerhet vid alla enheter/kliniker där forskningspersoner ska delta. Intyg från dessa ansvariga *ska* bifogas (se p. 9 bil. nr 9). Av intyget ska framgå att erforderliga ekonomiska, strukturella och personella resurser finns tillgängliga för att garantera forskningspersonernas säkerhet.

Olav Bengtsson, Divisionschef Barn och ungdomspsykiatri Stockholm

se bilaga 9

1:6 Ansökan/anmälan till andra myndigheter i vissa fall

(se p. 1:6 i Vägledning till ansökan)	Insänd	Datum
a)Vid klinisk läkemedelsprövning: Läkemedelsverket	☐	
b)Vid hantering av personuppgifter om genetiska anlag: Datainspektionen	☐	
c)Vid inrättande av biobank: Socialstyrelsen	☐	
d)Vid undersökning omfattande joniserande strålning: Strålskyddskommitté	☐	

2. Uppgifter om projektet

2:1 Sammanfattande beskrivning av forskningsprojektet

(Se p. 9 bil. nr 2 och p. 2:1 i Vägledning till ansökan)

Beskrivningen ska kunna förstås av nämndens samtliga ledamöter. Undvik därför terminologi som kräver specialkunskaper. Ange bakgrund och syfte för studien samt den/de vetenskapliga frågeställning (ar) som man söker svar på. Ange de viktigaste undersökningsvariablerna. Beskriv vilka kunskapsvinster projektet kan förväntas ge och betydelsen av dessa. Ange om det är en registerstudie, uppdragsforskning etc. För fackmän avsedd detaljerad information om forskningsplan/protokoll (program) *ska* bifogas som bilaga (se p. 9 bil. nr 2). För utformning av forskningsplan/protokoll se p. 2:1 i Vägledning till ansökan. Ange när datainsamlingen beräknas vara avslutad. En utförligare beskrivning av studiens genomförande *avsedd för lekmän* kan vid behov bifogas den för fackmän avsedda obligatoriska forskningsplanen.

1. Bakgrund

Personer med diagnosen tvångssyndrom/OCD (eng.: obsessive-compulsive disorder) lider av tvångstankar och tvångshandlingar som påverkar och försämrar fungerandet i vardagen. Mellan 0,5 % och 2 % av barn och ungdomar drabbas av tvångssyndrom. Symtomen medför ofta omfattande konsekvenser för barnet och dess familj och enligt Världshälsoorganisationen WHO räknas tvångssyndrom till de tio mest handikappande sjukdomar i världen. Eftersom symtomen ofta debuterar i barndomen är tidig upptäckt och behandling av central betydelse för sjukdomsförloppet och förebyggande av kronicitet.

Socialstyrelsen (2009) rekommenderar kognitiv beteendeterapi (KBT) som förstahandsval vid behandling av tvångssyndrom hos barn och ungdomar. KBT är effektiv för ca 70 % av patienterna och resulterar i en symtomreduktion på 40 till 64%. Även läkemedelsbehandling med selektiva serotonin återupptagshämmare (SSRI) är en effektiv behandling och rekommenderas främst i kombination med KBT.

Tyvärr diagnostiseras och behandlas tvångssyndrom sällan innan vuxen ålder och de geografiska skillnaderna i tillgänglighet till adekvat vård är stora. Dessutom blir identifierade fall sällan behandlade med effektiva metoder. För personer med tvång betyder detta är att tillståndet pågår obehandlat under manga år. I en studie till exempel var medeltiden från symtomdebut till insättning av effektiv behandling i snitt 17 år (Pinto et al., 2006).

Internetförmedlad KBT (IKBT) är ett nytt format att erbjuda patienter ökad tillgänglighet till effektiv psykologisk behandling. Innehållet i IKBT skiljer sig inte från traditionell KBT och baseras på kliniska metoder som har visat sig ha god evidens. I behandlingen arbetar patienten

med texter, övningar och hemuppgifter via en internetplattform. Patienten har flera gånger i veckan tillgång till en behandlare och får via mail och telefon återkoppling och stöd. Tidigare forskning har upprepade gånger visat att IKBT är effektiv för behandling av ångeststörningar och depression hos vuxna. Sedan 2007 erbjuder internetpsykiatrienheten vid Huddinge sjukhus IKBT för ångeststörningar och depression. En studie från 2012 från samma forskningsgrupp har visat att även OCD hos vuxna framgångsrikt kan behandlas med IKBT.

Vad gäller barn och ungdomar så är internetförmedlade behandlingar inte tillgängliga i Sverige i nuläget. Tidigare forskning har visat att IKBT är effektiv för barn med ångeststörningar. Däremot finns det inga publicerade resultat om IKBT för barn och ungdomar med tvångssyndrom.

Sammanfattningsvis är tvångssyndrom ett allvarligt psykiatriskt tillstånd som drabbar ungefär 1 barn av 100. KBT är en effektiv intervention för majoriteten av patienterna och rekommenderas som förstahandsval för behandling. Dock brister det på tillgänglighet av KBT inom vården. IKBT har visat sig vara effektiv för behandling av olika psykiatriska tillstånd hos barn och vuxna. Vi tror att IKBT har potentialen att öka tillgänglighet till effektiv behandling för tvångssyndrom hos barn och ungdomar.

2. Syfte

Det övergripande syftet med den planerade studien är att utveckla, testa och utvärdera IKBT för ungdomar med tvångssyndrom.

3. Frågeställningar

Är internetbaserad KBT vid OCD hos ungdomar en effektiv behandlingsmetod?

Hur ser långtidseffekten av IKBT ut?

Vilka variabler predicerar och modererar behandlingsutfall?

4. Metod

Den planerade studien är en pilotstudie där vi vill testa internetplattformen, användarvänligheten och effektiviteten av IKBT behandlingen. Effektiviteten kommer att mätas med olika kvalitativa psykometriska mått som kommer att bli jämförda via medelvärdsskillander före och efter behandlingen.

5. Förväntat vetenskaplig och klinisk nytta

Tvångssyndrom är ett vanligt psykiatriskt tillstånd hos barn och ungdomar som ger upphov till stort lidande och funktionsnedsättning för barnet och konsekvenser för barnets familj. KBT är en effektiv behandlingsmetod samtidigt som KBT behandling är en begränsad resurs inom vården som många i behov inte kommer i kontakt med.

Projektet förväntas att kunna ge svar på om IKBT är en effektiv metod för behandling av tvångssyndrom hos ungdomar. Kortsiktigt kommer resultaten från pilotstudien ge forskargruppen relevant information för planering av en större, kontrollerad studie. Långsiktigt tror vi att IKBT har potentialen att nå ut till fler patienter och kunna öka vårdens kapacitet att erbjuda KBT.

2:2 Vilken/vilka vetenskaplig (a) frågeställning (ar) ligger till grund för projektets utformning?

Om projektet kan karakteriseras som en hypotesprövning, ange den primära och eventuellt sekundära hypotesen. Hänvisning till mer detaljerad information för fackmän kan ske till bifogad forskningsplan/protokoll enligt punkt 2:1.

Är internetbaserad KBT vid OCD hos ungdomar en effektiv behandlingsmetod (definierat som signifikanta före / efter medelvärdesskillnader i relevanta mått)?

Hur ser långtidseffekten av IKBT ut (definierat som signifikanta medelvärdesskillnader mellan den initiala mätningen och 3, 6 och 12 månader efter behandlingen)?

Vilka variabler predicerar och modererar behandlingsutfall?

2:3 Redogör för resultat från relevanta djurförsök (Gäller klinisk behandlingsforskning)

Om djurförsök inte utförts ange skälen till detta.

ej applicerbart

2:4 Redogör översiktligt för undersökningsprocedur, datainsamling och datas karaktär

(Se p. 9 bil. nr 5 och p. 2:4 i Vägledning till ansökan)

Av beskrivningen ska framgå hur projektet planeras genomföras. Beskriv insamlade datas karaktär. Ange hur datas tillförlitlighet säkerställs (t.ex. kvalitetskontroll/monitorering). Vid enkäter och intervjuer ska beskrivas tillvägagångssätt och t.ex. frågors innehåll och hur slutsatser dras. Enkäter och skattningsskalor *ska* bifogas (se p. 9 bil. nr 5). För medicinsk forskning ska anges t.ex. typer av ingrepp, mätmetoder, antal besök, tidsåtgång vid varje försök, doser och administrationssätt för eventuella läkemedel och/eller isotoper, blodprovsmängd (även ackumulerad mängd vid multipla försök). Ange om och på vilket sätt undersökningsprocedur m.m. skiljer sig från klinisk rutin. Om en behandling studeras för första gången på människa ska detta framgå och relevanta säkerhetsrutiner beskrivas. Ange proceduren för att ge den eventuella behandling efter projektets slut, som kan erfordras. Ange procedur för insamling av biologiskt material. Redogör för datakällor och procedurer vid behandling av personuppgifter. För mer detaljerad information kan hänvisning ske till bilagd forskningsplan.

Datainsamling

Data från både ungdomar och föräldrar kommer att samlas in i form av självskattningsformulär samt strukturerade diagnostiska intervjuer. Mätningar görs före och omedelbart efter avslutad behandling. Uppföljningsdata inhämtas 3, 6 och 12 månader efter avslutad behandling. De

strukturerade intervjuerna genomförs före och efter behandling av psykologer eller läkare samt i vissa fall psykologkandidater under sista delen av sin utbildning som handleds av legitimerade psykologer. Intervjuerna genomförs live vid bedömming och direkt efter behandling och över telefon vid uppföljning. Självskattningsformulären kommer att fyllas i på internet. För att kunna fylla i självskattningsformulären på ett säkert sätt får varje deltagare en personlig inloggningskod.

Självskattningsformulären undersöker ungdomarnas tvångsproblematik och screenar för övriga psykiatriska diagnoser. Föräldrarna fyller dels i formulär angående ungdomens problematik och funktionsnivå men även kring sitt eget mående med avseende på ångest, depression och tvång samt anpassningar som familjen har gjort till ungdomens tvångsproblematik.

Det primära utfallsmåttet är den semistrukturerade intervjun Children's Yale Brown Obsessive Compulsive Scale, CY-BOCS som mäter svårighetsgrad av tvångshandlingar och tvångstankar hos barn och ungdomar.

För att säkerställa diagnosen tvångssyndrom och annan psykopatologi kommer Mini International Neuropsychiatric Interview for Children and Adolescents, MINI-KID användas. MINI-KID är en halvstrukturerad klinisk screeningintervju.

Sekundära utfallsmått är: Clinical Global Impression – Severity, CGI-S (klinikerskattad grad av tvångsproblematik), Clinical Global Impression - Improvement, CGI-I (klinikerskattad grad av förbättring efter behandling), Children's Global Assessment Scale (CGAS, klinikerskattad allmän funktionsnivå), Children's Obsessional Compulsive Inventory Revised, (CHOCI-R, själv- och föräldraskattad grad av tvångsmässighet), Child Obsessive-Compulsive Impact Scale – Revised (COIS-R, själv- och föräldraskattad grad av i vilken utsträckning tvången hindrar ungdomen i vardagen), Spence Child Anxiety Scale – Child and Parent version (SCAS/P, själv- och föräldraskattad ångestsymtom, inom olika områden), Child Depression Inventory (CDI, självskattning depression), Strengths Difficulties Questionnaire (SDQ, själv och föräldraskattad psykopatologi och styrkor), Family Accommodation Scale, Parent-Report (FAS-PR, föräldraskattning av anpassningar som familjen gjort till ungdomens tvångsproblematik).

Föräldrarnas psykopatologi kommer att mätas med följande självskattnigsformulär: PHQ-9 (depression), GAD-7 (generell ångest), Obssessive Compulsive Inventory – Revised (OCI-R, tvångstankar och tvångshandlingar).

Datainsamling ang. biverkningar av behandlingen. Deltagarna kommer under behandlingen gång få svara skriftligt på öppna frågor om eventuella biverkningar av behandlingen.

Se bilaga 5 för en utförlig redovisning av använda mått.

Procedur

Projektet annonseras framförallt via media men även via BUP, skolhälsovård och primärvård.

Inklusion av deltagare kommer att ske i två steg: 1. telefonintervju, 2. diagnostisk intervju.

1. Telefonintervju

Initialt görs en telefonintervju med intressenter ang. allmänna inklusionskriterier för studien och en initial screening för tvångssyndrom.

2. Diagnostisk intervju

Ungdomar som inte exkluderats efter telefonintervju kommer till en face-to-face diagnostisk intervju som består av MINI-KID (i syfte att fastställa diagnosen tvångssyndrom) och CY-BOCS (i syfte att bedöma grad av tvång).

Individer som uppfyller inklusionskriterierna (se 3.1) blir erbjudna deltagande i studien och fyller i informerat samtycke. Därefter fyller deltagare (både föräldrar och ungdom) i självskattningsformulär på internet.

Intervention

Deltagare som fyllt i självskattningar får tillgång till IKBT interventionen via personliga login. Interventionen består av internetbaserad material i form av ett antal olika kapitel. Interventionen är indelad i en ungdomsdel och en föräldradel med information som är specifik för ungdomen och föräldern, men som i stort följer samma teman i behandlingen. Behandlingen är indelad i 4 olika faser: Analys, exponering, avslut och booster. Analysfasen består av psykoedukativa texter, förklaringsmodeller för tvångssyndrom ges och ungdomen/föräldern får arbeta med förståelsen och analysen av sitt eget tvångssyndrom. I exponeringsfasen jobbar ungdomen och föräldern aktivt med att minska på tvångshandlingar och tvångstankar, stegvis och strukturerat. I avslutsfasen handlar det om att befästa framsteg och planera vidmakthållande åtgärder efter behandlingens avslut. Boosterfasen innebär att deltagare kontaktas igen av klinikern 3, 6 och 12 månader efter avslutad ordinarie behandling för att upprätthålla framsteg och vid behov lösa problem som uppstått efter avslut av ordinarie behandling. Den ordinarie behandlingens längd (faserna analys, exponering, avslut) är 12 veckor.

Veckovis mätning av tvångsmässighet: föräldrar och ungdomar kommer varje vecka fylla in självskattningar av tvångsmässighet i formuläret CHOCI-R.

Säkerhetsrutiner

Kliniker som i kontakt med deltagare får information om faktiska eller misstänkta säkerhetsrisker för deltagare kontaktar föräldrar omgående via telefon för att vidta lämpliga åtgärder. Säkerhetsrisker i den bemärkelsen definieras som situationer där det finns risk för deltagares eller annans hälsa eller välmående, exempelvis suicidrisk eller avsikt att skada annan person. Studiekoordinatorn (Fabian Lenhard) informeras vid upptäckt av säkerhetsrisker. Ifall fortsatt deltagande i studien ej bedöms adekvat utifrån säkerhet exkluderas ungdomen från studien. Vid behov av andra insatser, vård eller omhändertagande ges motsvarande kontaktinformation (exempelvis till BUPs öppen- eller slutenvård vid ungdomspsykiatriska behov).

2:5 Redogör för om insamlat biologiskt material kommer att förvaras i en biobank

(Se p. 2:5 i Vägledning till ansökan)

Med biobank avses biologiskt material från en eller flera människor som samlas och bevaras tills vidare eller för en bestämd tid och vars ursprung kan härledas till den eller de människor från vilka materialet härrör. Redogör för var och hur prover som ska sparas förvaras, kodningsprocedurer och villkor för utlämnande av prover. Ange huvudman för biobanken.

ej applicerbart

2:6 Dokumentation, dataskydd och arkivering (Se p. 2:6 i Vägledning till ansökan)

Redogör för hur undersökningsprocedurer och eventuella ingrepp dokumenteras. Ange om band- och videoinspelningar används. Om materialet ska kodas, ange proceduren, vem som förvarar kodlistor/kodnycklar och vem eller vilka som har tillgång till dem, var och hur länge de förvaras samt om materialet kommer att anonymiseras eller förstöras. Redogör för vilken tillgänglighet datamaterialet har och hur det förvaras samt hur erforderligt sekretesskydd erhålls.

All information kommer att sekretesskyddas och deltagaren tilldelas en personlig kod i studien. Deltagarnas personuppgifter och kodnyckeln som länkar dem till deras personliga kod kommer att förvaras i en datafil skyddad med dubbla autenticieringskrav på en säker server. Endast ansvariga för projektet har tillgång till dessa uppgifter. Filen kommer även att krypteras och lösenordskyddas för att uppfylla krav på hantering av personuppgifter enligt personuppgiftslagen.

Data från frågeformulär kommer att registreras i avidentifierad form. Video- och ljudinspelningar från bedömningsintervjuerna, om sådana spelats in, kommer att förvaras i låst journalskåp.

Personuppgifter som kommer att sparas i internetplattformen är deltagarens mailadress och telefonnummer. Anledningen till detta är att det ger möjlighet till en säker inloggning via s.k. dubbel autentisiering (likt den proceduren som används t.ex. vid internetbanking eller av Vårdguidens internettjänster).

För att säkerställa att dessa personuppgifter lagras i enlighet med Socialstyrelsens riktlinjer för patientuppgifter (2 kap. 5 § SOSFS 2008:14) installeras ett s.k. SSL-certifikat som krypterar trafiken mellan plattformen och andra servrar. Detta är i enlighet med Socialstyrelsens riktlinjer om ”stark autentisering” av användare. Detta innebär att det blir det mycket svårt för obehöriga att ta sig in i plattformen.

Vid Internetpsykiatrienheten (Stockholms läns landsting), där flera studier av internetbehandling har genomförts och där man behandlar med IKBT i ordniarie vård, används samma teknik för att lagra och skydda personuppgifter i syfte att kunna skicka påminnelser via mail och sms.

2:7 Redogör för tidigare erfarenheter (egna och/eller andras) av den använda proceduren, tekniken eller behandlingen

Särskilt angeläget är att redovisning av risker för komplikationer görs tydliga och i förekommande fall med angivande av relevanta publikationer. Vid nya behandlingar av patienter, t.ex. med läkemedel, bör anges hur många patienter (med aktuell eller annan åkomma) som tidigare erhållit föreslagen behandling, läkemedelsdosering (eller annan dosering) samt hur långa behandlingsperioder som studerats.

KBT är rekommenderad som förstahandsval för behandling av tvångssyndrom hos barn och ungdomar enligt Socialstyrelsen. Metoden är välbeforskad och har god evidensstöd (se även kunskapsöversikt i projektplanen, bilaga 2).

Internetförmedlad KBT är en väldokumenterad, evidensbaserad behandlingsform för olika psykiatriska tillstånd hos vuxna. 2012 kom den första randomiserade, kontrollerade studien för tvångssyndrom hos vuxna, med god effekt (E. Andersson, 2012).

På Psykiatri Sydvästs internetpsykiatrienhet, Huddinge sjukhus, har man sedan 2007 erbjudit IKBT i ordinarie vård för vuxna patienter. Där har även genomförts ett flertal studier på olika tillstånd och bred kompetens finns både kring kliniska rutiner och genomförande av vetenskapliga arbeten inom området. Christian Rück, PhD, överläkare på internetpsykiatrienheten och forskningsgruppsledare och Erik Andersson, psykolog och doktorand, ingår i forskningsgruppen kring den planerade studien. Med i projektgruppen finns även professor Gerhard Andersson från Institutionen för beteendevetenskap och lärande vid Linköpings Universitet och Karolinska Institutet som har lång erfarenhet av att bedriva forskning på internetbaserad behandling.

Vår forskningsgrupp, under ledning av Eva Serlachius, PhD, överläkare, har erfarenhet av flera pågående studier inom IKBT området: en avslutad pilotstudie om IKBT behandling av barn med

ångesttillstånd (Dnr. 2010/1993-31/4), en pågående randomiserad, kontrollerad studie inom samma fält (Dnr. 2011/2027-31/5) samt en pågående pilotstudie om behandling av IBS.

De forskare som är kopplade till projektet har alla lång erfarenhet av kliniskt arbete med psykiatriska patienter och det finns bred kompetens av behandling av tvångssyndrom.

3. Uppgifter om forskningspersoner

3:1 Hur görs urvalet av forskningspersoner? (Se p. 9 bil. nr 3 och p. 3:1 i Vägledning till ansökan)

Med forskningsperson avses en levande människa som forskningen avser. Ange urvalskriterier (inklusion och exklusion). Redogör för på vilket sätt forskaren kommer i kontakt med/får kännedom om lämpliga forskningspersoner. Om annonsering sker, *ska* annonsmaterialet insändas som bilaga (se p. 9 bil. nr 3). Om t.ex. barn eller personer som tillfälligt eller permanent inte är kapabla att ge ett eget informerat samtycke ska ingå i projektet, ska detta särskilt motiveras. Om vissa grupper utesluts från deltagande i projektet ska detta särskilt motiveras.

Projektet annonseras framförallt via media men även via BUP, skolhälsovård och primärvård (för annonsmaterial se bilaga 3).

Inklusionskriterier:

- uppfyller kriterier för diagnosen tvångssyndrom (enligt DSM-IV)
- totalpoäng på > 15 på CY-BOCS
- mellan 12 och 17 år
- läser och skriver svenska
- daglig tillgång till internet
- en förälder har möjlighet att delta i behandlingen
- vid medicinering: en stabil dos under de senaste 6 veckorna innan den initiala bedömningen
- underskrivet informerat samtycke

Exklusionskriterier

- har en av följande diagnoser: autismspektrumstörning, psykossjukdom eller bipolär sjukdom
- bedöms suicidbenägen
- pågående substansmissbruk
- svårigheter att förstå eller använda IKBT materialet / upplägget (ffa. språkliga svårigheter)

☐ genomfört KBT behandling för tvångssyndrom under det gångna året (minst 5 sessioner med exponeringsbaserad behandling)

☐ pågående psykologisk behandling mot ångest eller tvångssyndrom

3:2 Ange relationen mellan forskare/försöksledare och forskningspersonerna

☒ Behandlare (t.ex. läkare, psykolog, sjukgymnast) - forskningsperson (t.ex. patient, klient)

☐ Kursgivare (lärare) - student

☐ Arbetsgivare - anställd

☐ Annan relation som kan tänkas medföra risk för påverkan. Beskriv:

3:3 Redogör för det statistiska underlaget för studiepopulationens (-ernas)/ undersökningsmaterialets (-ens) storlek (Se p. 3:3 i Vägledning till ansökan)

Redovisa statistisk styrka, så kallad ”power”- beräkning eller redovisa motsvarande överväganden som tydliggör studiens möjligheter att besvara frågeställningarna.

För att uppnå 80 %ig power på studiens primärmått räknar vi med en grupp på n = 27 deltagare vid måttlig effektstyrka d = 0,5, (signifikansnivå p = .05).

3:4 Kan forskningspersonerna komma att inkluderas i flera studier samtidigt eller i nära anslutning till denna? I så fall, vilken typ av forskning?

(Se p. 3:4 i Vägledning till ansökan)

ej aktuellt

3:5 Vilket försäkringsskydd finns för de forskningspersoner som deltar i projektet?

Det åligger forskningshuvudmannen att kontrollera att det finns försäkring som täcker eventuella skador som kan uppkomma i samband med forskningen.

Ingen separat försäkring kommer att tecknas.

3:6 Vilken ekonomisk ersättning eller andra förmåner utgår till de forskningspersoner som deltar i projektet och när betalas ersättningen ut? Utförligare beskrivning kan lämnas i bilaga.

(Se p. 9 bil. nr 11 och p. 3:6 i Vägledning till ansökan)

Ersättning för obehag och besvär. Ange belopp (före skatt):	2 biobiljetter á 100 kr	
Ersättning för förlorad arbetsinkomst	☐ Ja	☒ Nej
Reseersättning	☐ Ja	☒ Nej
Befrielse från kostnader för läkemedel	☐ Ja	☒ Nej
Befrielse från andra kostnader. Vilka?		
Andra förmåner. Vilka?		
När betalas ersättningen ut?	vid uppföljning (efter genomgången behandling)	
Ingen ersättning betalas ut	☐	

4. Information och samtycke

4:1 Proceduren för och innehållet i den *information* som lämnas då forskningspersoner tillfrågas om deltagande

(Se p. 9 bil. nr 4 och Vägledning till forskningspersonsinformation.)

Enligt 16 § lag (2003:460) om etikprövning av forskning som avser människor ska forskningspersonen informeras om den övergripande planen för forskningen, syftet med forskningen, de metoder som kommer att användas, de följder och risker som forskningen kan medföra, vem som är forskningshuvudman, att deltagande i forskningen är frivilligt och forskningspersonernas rätt att när som helst avbryta sin medverkan. Beskriv hur och när information ges och vad den innehåller. Ange vem som informerar. Normalt ska en kortfattad och lättförståelig skriftlig information ges. Denna skriftliga information *ska* bifogas ansökan (se p. 9 bil. nr 4). Om ingen eller ofullständig information ges, måste skälen för detta noggrant anges.

Projektet kommer att annonseras i media. I annonstexten finns internetadressen till studiens hemsida. På denna sida finns information om vad studien innebär/kräver av familjerna, kontaktuppgifter till projektet samt information om hur man anmäler sitt intresse. Information finns riktad till både ungdomar och föräldrar. Vid anmälnings- och screeningsamtalet på telefon ges intressenter möjlighet att ställa frågor. För ungdomar sker muntlig och skriftlig information, anpassat till deras nivå, och möjlighet att ställa frågor även vid det tillfälle då de kommer för bedömning.

4:2 Hur och från vem inhämtas *samtycke*? (Se Vägledning till forskningspersonsinformation)

Beskriv proceduren; vem som frågar, när detta sker och hur samtycket dokumenteras. Utförlig redovisning är särskilt viktig då barn eller personer med nedsatt beslutskompetens ingår i studien, likaså vid studier av en grupp/grupper, t.ex. skolklasser, föreningar, organisationer, företag, kyrkosamfund, församlingar eller grupper som interagerar inom sociala medier.

Informerat samtycke samlas in skriftligt från båda vårdnadshavare och ungdomen efter att de fått möjlighet att ställa frågor vid live-bedömningen. För att delta i studien måste även samtycke enligt Personuppgiftslagen (PUL) ges (se bilaga 4).

5. Forskningsetiska överväganden

5:1 Redogör för alla risker som deltagandet kan medföra

Dessa kan vara t.ex. fysisk eller psykisk skada, smärta, obehag eller integritetsintrång på kort eller lång sikt. Ange vilka åtgärder som har vidtagits för att förebygga riskerna som nämns ovan samt vilken beredskap som

finns för att hantera sådana komplikationer. Ange vilka/de metoder som kommer att användas för att efterforska, registrera och rapportera oönskade händelser.

Behandlingen syftar till att reducera symtomgrad av tvångsmässighet. Den kognitiva beteendeterapeutiska behandlingsmodellen innebär att patienten får utsätta sig för kortsiktigt obehagliga situationer i syfte att uppnå långsiktigt symtomdämpade effekter. För att patienten inte ska uppleva detta integritetskränkande läggs stor vikt på att patienten förstått vad behandlingen innebär och att förbereda exponeringsövningar noga.

Inga negativa effekter bedöms föreligga av behandlingsprogrammet eller bedömningsförfarandet i sig. En omfattande bedömning och uppföljning kan upplevas negativ men är en nödvändig del i utvärderingen av behandlingen. Dessutom kan både bedömningsförfarandet och behandlingen i sig, liksom i all psykologisk behandling, upplevas som integritetsinkräktande. För att förebygga detta kommer information om hur bedömning och behandling går till att ges på projekthemsidan.

Att bli exkluderad från studien kan upplevas som negativt. För att förebygga detta finns tydlig information om exklusions- och inklusionskriterier på projektets hemsida. All exkludering sker via telefon med personlig feedback och, om aktuellt, med hänvisning till adekvat vård.

Vidare kan deltagarna uppleva oro kring datasäkerhet vilket också kommer att förebyggas genom tydlig information om hur hantering av data sker. Sedvanlig sekretess gäller och all information kommer att förvaras avkodat och inlåst.

Familjen har närsomhelst rätt att avbryta sitt deltagande i studien. Då studien sker utanför reguljär vård kommer ett val att inte delta i studien inte att påverka de vanliga vårdkontakterna. Ungdomen kommer att infomeras muntligt och skriftligt om studien och tydligt infomeras om rätten att närsomhelst säga nej till deltagande.

5:2 Redogör för möjlig nytta för de forskningspersoner som ingår i projektet (gäller särskilt behandlingsforskning)

Behandlingen består av evidensbaserade metoder för behandling av tvångssyndrom. Vi bedömer det som sannolikt att deltagare kan profitera av interventionen och uppleva symtomlindring som effekt av behandlingen. Nyttan med internetlevererad behandling är även att behandlingen genomförs hemma och att ungdom och föräldrar inte behöver ta sig till en mottagning. Då behandlingen också riktar sig till föräldern och dess roll är en möjlig effekt att föräldern kommer att lära sig strategier för att bättre kunna bemöta ungdomens oro samt att hjälpa ungdomen att bygga upp mer funktionella beteendemönster.

5:3 Identifiera och precisera om eventuella etiska problem (fördelar/nackdelar) kan uppstå i ett vidare perspektiv genom projektet (Se p. 5:3 i Vägledning till ansökan)

Här kan redovisas om exempelvis vissa grupper (andra än de forskningspersoner som ingår i forskningsprojektet) kan komma att utpekas/få hjälp som ett resultat av studien.

En potentiell etisk risk med studieupplägget är att studiedeltagarna kommer att vara en förselekterad grupp. Rekrytering till vetenskapliga studier via annonser kan tänkas attrahera en viss socioekonomisk grupp och att det därför blir en snedfördelning i urvalet. Ytterligare risk för denna snedfördelning uppstår igenom att deltagande i studien förutsätter goda språkkunskaper i svenska. Internetförmedlad KBT måste därför ses som ett komplement till ordinarie behandling i ett vidare perspektiv, inte som ett substitut.

I det långa loppet skulle denna forskningslinje leda till att erbjuda fler ungdomar KBT för tvångssyndrom och att göra KBT tillgängligt utan att behöva ta sig till en mottagning. Detta skulle framför allt vara en stor nytta för de familjer som bor långt ifrån specialiserad psykiatrisk vård.

6. Redovisning av resultaten

6:1 Hur garanteras forskningshuvudmannen och medverkande forskare tillgång till data (anges vid t.ex. uppdragsforskning) och vem ansvarar för databearbetning och rapportskrivning?

(Se p. 6:1 i Vägledning till ansökan)

Deltagarna i forskargruppen ansvarar gemensamt för databearbetning och rapportskrivning.

6:2 Hur kommer resultaten att göras offentligt tillgängliga? Kommer studien att insändas för publicering i tidskrift eller publiceras på annat sätt? (Se p. 6:2 i Vägledning till ansökan)

Ange i vilken form resultaten planeras offentliggöras samt tidsplan för detta.

Studien och resultaten används i en doktorsavhandling. Resultaten publiceras i nationella och internationella vetenskapliga tidskrifter. Resultaten redovisas på internationella och nationella kongresser.

6:3 På vilket sätt garanteras forskningspersonernas rätt till integritet när materialet offentliggörs/publiceras?

Redovisas resultat på statistisk gruppnivå? Beskriv procedurer eller metoder för avidentifiering/anonymisering.

Resultaten kommer huvudsakligen att redovisas på gruppnivå. Om data presenteras på individnivå kommer dessa att vara avidentifierade.

7. Redovisning av ekonomiska förhållanden och beroendeförhållanden

Redovisning enligt punkterna 7:1-7:3 syftar till att tydliggöra alla direkta eller indirekta förhållanden, som kan tänkas påverka forskarens relation till forskningspersonerna (vid t.ex. informations-, samtyckes-, genomförandeprocedurer).

7:1 Vid uppdragsforskning

Ange uppdragsgivaren t.ex. ett företag (vid klinisk läkemedelsprövning eller prövning av andra nya produkter), en organisation eller en myndighet.

Namn: Kontaktperson:

Adress: Telefon/mobiltelefon:

Ange uppdragsgivarens relation till forskningshuvudmannen/medverkande forskare, t.ex. anställningsförhållande

ej applicerbart

7:2 Redovisa eventuella ekonomiska överenskommelser med uppdragsgivare eller andra finansiärer (namn, belopp)

Vid klinisk läkemedelsprövning bör hänvisning ske till ingånget avtal med sjukvårdshuvudmannen. Liknande överenskommelser kan förekomma vid annan uppdragsforskning och bör redovisas på samma sätt. Separata överenskommelser med den/de som ska genomföra forskningen ska redovisas. Belopp som kommer att erhållas för studien/ersättning till kliniken/genomföraren, vad ersättningen bör täcka och ev. belopp som erhålls per forskningsperson, bör också anges här.

ej applicerbart

7:3 Redovisa forskningshuvudmannens, huvudansvarig forskares och medverkande forskares egna intressen

Här redovisas t.ex. aktieinnehav, anställning, konsultuppdrag i finansierande företag, eget företag som kan få (direkt eller indirekt) ekonomisk vinst av forskningen.

ej applicerbart

8. Undertecknande

Behörig företrädare för sökande forskningshuvudman enligt p. 1:2.

Ort: Datum:

Signatur: ____

Namnförtydligande: Olav Bengtsson

Tjänstetitel: Divisionschef, Barn och ungdompsykiatrin Stockholm

Undertecknad forskare som genomför projektet (kontaktperson) enligt p. 1:3 intygar härmed att forskningen kommer att genomföras i enlighet med ansökan.

Ort: Datum:

Signatur: ____

Namnförtydligande: Eva Serlachius

Tjänstetitel: Överläkare, Med. Dr.

9. Förteckning över bilagor (Se p. 9 i Vägledning till ansökan)

Dokument som, i tillämpliga fall, ska bifogas *om inte motsvarande information finns i blanketten* har markerats med x. Markera de bilagor som skickas in med denna ansökan.

Insänd med ansökan	Bil nr	Beskrivning	Klinisk läkemedels-prövning	Annan forskning
☒	1	Deltagande forskningshuvudmän och medverkande forskare (kontaktpersoner) vid forskning där mer än en forskningshuvudman deltar. Se p. 1:4	x	x
☒	2	För fackmän avsedd forskningsplan, vid behov även för lekmän avsedd bilaga. Se p. 2:1 och Vägledning till forskningsplan/forskningsprotokoll (program)	x	x
☒	3	Annonsmaterial för rekrytering av forskningspersoner. Se p. 3:1 och i Vägledning till ansökan p. 3:1	x	x
☒	4	Skriftlig information till dem som tillfrågas. Se p. 4:1 och Vägledning till forskningspersonsinformation och (i förekommande fall) separat samtyckesformulär	x	x
☒	5	Enkät, frågeformulär. Se p. 2:4	x	x
☐	6	Gemensam EU blankett (gäller fr.o.m. den 1 maj 2004), gäller även vid ändring. För information se Läkemedelsverkets hemsida, www.lakemedelsverket.se	x	
☐	7	Sammanfattning av protokollet på svenska	x	
☐	8	Prövarhandbok alt. bipacksedel/produktresumé/IB	x	
☒	9	Intyg från verksamhetschef/motsv. om resurser för forskningspersonernas säkerhet. Se p. 1:5 och förslag till utformning av resursintyg i Vägledning till ansökan p. 1:5	x	x
☒	10	CV för forskare (samma som p. 1:3) med huvudansvar för genomförandet, redovisa forskarens (- arnas) kompetens av relevans för studien. Se Vägledning till ansökan p. 1:3	x	x
☐	11	Beskrivning av ersättning till forskningspersoner. Se p. 3:6 och i Vägledning till ansökan p. 3:6	x	x

Övriga bilagor som bifogas ansökan:
