# Supplementary material for: Internet-Delivered Cognitive Behavior Therapy for Adolescents with Obsessive-Compulsive Disorder: An Open Trial
Source: PLoS One. 2014 Jun 20;9(6):e100773. doi: 10.1371/journal.pone.0100773 (PMC4065049; doi:10.1371/journal.pone.0100773)
Supplement: Protocol S2 — Trial Protocol (English). (PDF) [file pone.0100773.s005.pdf]

Study protocol

Internet-delivered Cognitive Behavioural Therapy for Adolescents with Obsessive-Compulsive Disorder: a pilot study

1. Background

Obsessive-Compulsive Disorder (OCD) is characterized by obsessive thinking and/or compulsive behaviours (American Psychiatric Association, 1994). OCD affects between 0,5 (Flament et al., 1988) and 2 % of children (Angst et al., 2004; Valleni-Basile et al., 1994). Paediatric OCD is commonly associated with severe impairments in academic, social and family functioning (Valderhaug & Ivarsson, 2005) and increases the risk of future mental health problems in adulthood (Wewetzer et al., 2001). As OCD frequently debuts in childhood, early intervention is crucial for long-term outcome and prevention of chronicity (Micali et al., 2010). Unfortunately, OCD is rarely adequately treated until adulthood as a result of combined factors, such as poor diagnostic detection, ineffective treatments and patients delay (Pinto, Mancebo, Eisen, Pagano, & Rasmussen, 2006). Availability of proper assessment and treatment differ both locally and regionally (Goodwin, Koenen, Hellman, Guardino, & Struening, 2002) and a majority of cases are not detected by health care (Wahl et al., 2010). In addition, recognized cases of OCD are seldom treated with effective psychological interventions (Valderhaug, Götestam, & Larsson, 2004). As a result, many patients' suffering is prolonged. For instance, one study of a clinical sample found that the mean time from OCD symptom onset (12 years of age) to receiving effective treatment was on average 17 years (Pinto et al., 2006).

1.1. Treatment of OCD

Cognitive behaviour therapy (CBT) is recommended as the first-line treatment for paediatric OCD in the national guidelines of the Swedish National Board of Health and Welfare (Socialstyrelsen, 2009). Meta-analyses have repeatedly shown that CBT is an effective treatment for paediatric OCD with remission rates about 70% and an average symptom reduction of 40 - 64% (Abramowitz, Whiteside, & Deacon, 2000; Reynolds, Wilson, Austin, & Hooper, 2012; Watson & Rees, 2008).

Pharmacological treatment with serotonin reuptake inhibitors (SRIs) is an alternative or supplement to CBT. SRIs are an effective treatment for the condition, with a reduction of symptoms of 30 – 40% (Geller et al., 2003; Watson & Rees, 2008).

1.1.1. Internet-delivered CBT and treatment of anxiety disorders

Internet-delivered CBT (ICBT) is a new way of increasing CBT accessibility and treatment capacity. The treatment content is not different from regular CBT but the way of delivery. In ICBT, the patient works with web-based treatment material and homework assignments that are examined by a therapist via email and telephone. ICBT have been found to be effective in the treatment of adults with depression and anxiety disorders (G. Andersson et al., 2005; Bergström et al., 2010; Hedman, Andersson, Ljótsson, et al., 2011; Spek et al., 2007). Studies have not only proved

internet-based treatments' efficiency compared to waitlist control, but have also been able to show results comparable to standard face-to-face treatment (Bergström et al., 2010; Hedman, Andersson, Ljótsson, et al., 2011). The research teams of Rück, G. Andersson and Mataix-Cols have published several controlled studies on ICBT for adults (G. Andersson, 2009; Hedman, Andersson, Andersson, et al., 2011; Hedman, Andersson, Ljótsson, et al., 2011; Schneider, Mataix-Cols, Marks, & Bachofen, 2005). Andersson et al recently published the first controlled trial on ICBT for adults with OCD (E. Andersson et al., 2012). The Internet psychiatry unit at Karolinska University Hospital has since 2007 successfully implemented ICBT for depression and anxiety disorders within public health care.

Internet-delivered treatment for children and adolescents: ICBT has been found to be effective for various anxiety disorders for children aged 7 to 14 years (S. March, Spence, & Donovan, 2009). Though, to our knowledge there is no published data on ICBT and paediatric OCD.

To summarize, paediatric OCD is a serious psychiatric disorder that commonly is associated with significant impact on the individual's well-being and quality of life. CBT is effective for the majority of cases and is recommended as the first-hand intervention for treatment. Unfortunately accessibility to CBT is limited. Internet-delivered interventions have the potential to increase availability and range of effective psychological treatment for children and adolescents. ICBT has been shown to be effective in adults with various psychiatric conditions and children with anxiety disorders.

In Sweden, internet-based treatments for adolescents are not yet available within public health care. Our research group is currently studying an ICBT-intervention for anxiety disorders among Swedish children 8 to 12 years. A technical platform and ICBT protocol has been developed (www.bup.se/bip) and publication of first results are expected in 2013. We now want to expand ICBT interventions to adolescents with OCD.

2. Objective

The overall purpose is to develop and test the feasibility and efficacy of ICBT for paediatric OCD. In this pilot we want to test the technical platform, treatment manual and its efficacy. The results in the pilot study will guide us in the proper practical and statistical planning of a randomized controlled trial.

3. Method

3.1. Subjects

Inclusion of participants compromise the following criteria:

- a primary diagnosis of OCD as defined by DSM-IV TR
- a total score of above 15 on the CY-BOCS
- age between 12 and 17 years
- ability to read and write Swedish
- daily access to the internet
- a parent that is able to co-participate in the treatment
- Participants on psychotropic medication must have been on a stable dose for the last 6 weeks prior to baseline assessment
- signed informed consent

Subjects will be excluded if:

- diagnosed autism spectrum disorder, psychosis or bipolar disorder
- suicidal ideation
- ongoing substance dependence
- subject not able to read or understand the basics of the ICBT self-help material
- completed CBT for OCD within last 12 months (defined as at least 5 sessions CBT including exposure and response prevention)
- ongoing psychological treatment for OCD or another anxiety disorder

3.2. Measures

The measures in this study have been chosen with respect to diagnostic accuracy as well as earlier findings on moderators and predictors of pediatric OCD treatment (Ginsburg, Kingery, Drake, & Grados, 2008).

1.1.2. Diagnostic and symptom severity measures

Mini International Neuropsychiatric Interview for Children and Adolescents, MINI-KID (D V Sheehan et al., 1998) is a short structured diagnostic interview for DSM-IV and ICD-10 psychiatric disorders in children and adolescents. MINI-KID is a reliable diagnostic instrument and is validated against the widely used MINI-KID-PL interview (D V Sheehan et al., 2010).

Children's Yale Brown Obsessive Compulsive Scale, CY-BOCS (Scahill et al., 1997). CY-BOCS is a semi-structured clinician administered interview and considered the golden standard in assessment

of OC symptom severity in children and adolescents. CY-BOCS has high internal consistency, good to excellent inter-rater agreement and correlates significantly with self-ratings of OC symptoms (Scahill et al., 1997). CY-BOCS will be the primary outcome measure.

Clinical Global Impression – Severity, CGI-S (National Institute of Mental Health, 1985). CGI-S is a brief clinician rating of symptom severity. Ratings on the seven graded scale range from 1 = "no symptoms" to 7 = "extreme severe". CGI-S correlates highly with the CY-BOCS total score (r = .75, Storch et al., 2004).

Clinical Global Impression - Improvement, CGI-I (Guy, 1976). CGI-I is a brief clinician rating of the patients symptom severity change relative to the basement assessment. The seven graded scale ranges from 1 = "very much improved" to 7 = "very much worse".

Children's Global Assessment Scale (CGAS, Shaffer et al., 1983). CGAS is a widely accepted instrument to quantify the overall level of functioning in children and adolescents. The 100-point scale is clinician rated and has demonstrated to have good discriminant and concurrent validity (Shaffer et al., 1983) as well as moderate inter-rater reliability in naturalistic settings (Lundh, Kowalski, Sundberg, Gumpert, & Landén, 2010).

1.1.3. Self-report measures

All self-report measures will be internet-administrated.

The *Children's Obsessional Compulsive Inventory Revised,* (CHOCI-R, Shafran et al., 2003) is a self- and parent-report measure of paediatric OCD symptom severity. The CHOCI-R has good internal consistency and correlates significantly with the CY-BOCS (Uher, Heyman, Turner, & Shafran, 2008).

Child Obsessive-Compulsive Impact Scale – Revised (COIS-R, Piacentini, Peris, Bergman, Chang, & Jaffer, 2007) is a self-report scale of OCD symptom impact on everyday life. COIS-R is available as a youth and parent version.

Spence Child Anxiety Scale – Child and Parent version (SCAS/P, Spence, 1998) will be used as a child and parent self-report measure of anxiety related psychopathology. The 45 items of the SCAS/P result in 6 subscales: panic attacks and agoraphobia, separation anxiety, physical injury fears, social phobia, obsessive-compulsive and generalized anxiety.

Symptom severity of depression in the adolescent will be assessed with *Child Depression Inventory* (CDI, Kovacs, 1985).

Strengths Difficulties Questionnaire (SDQ, Goodman, 1997) is a widely used self- and parent-report behavioral screening questionnaire with six subscales: emotional problems, conduct

problems, hyperactivity-inattention, peer problems, prosocial behavior.

Patient Health Questionnaire PHQ-9 (Kroenke, Spitzer, & Williams, 2001) is a brief self-report measure of depressive symptoms in adults. PHQ-9 will be used to measure parental depressive symptoms.

GAD-7 (Spitzer, Kroenke, Williams, & Löwe, 2006) is a brief self-report measure of symptoms of general anxiety in adults. GAD-7 will be used to measure symptoms of anxiety in the parents.

Obsessive Compulsive Inventory – Revised (OCI-R, Foa et al., 2002) is a reliable and valid 18-item self-report scale for screening of OCD in adults and will be used to measure parental OC symptoms of both parents.

Family Accommodation Scale, Parent-Report (FAS-PR, Flessner et al., 2009). Originally a clinician-rated instrument (Calvocoressi et al., 1999), the parent-report version of the FAS consists of 12 items focusing on accommodation behaviors of parents with a child with OCD. Parental and family accommodation in OCD is expected to be a predictor of treatment outcome (Garcia et al., 2010). FAS-PR will be filled out by both parents.

Adverse events assessment. During the treatment phase of the study participants will be asked to fill in a web-based form on the occurrence of any adverse events as a reaction to or consequence of the treatment. Adverse events frequency will be reported in the publication of the study.

In the self-report procedure parents will even be asked to answer questions about parental educational level and gross household income, ethnicity of adolescent and parents and other demographic data. Moreover, participants will be asked about prior treatment history (e.g. previous CBT or medication) and response to previous treatments. Table 1 gives an overview of the measures that will be used in the study. Attachment 1 displays an overview of which measures are used when in the study.

Table 1: List of measures

Category	Clinician assessed	Self-report adolescent	Self-report parent
**Demographics**	Age<br>Sex<br>Family structure		Educational level<br>Gross household income<br>Ethnicity
**OC symptom severity**	CY-BOCS<br>CGI-S<br>CGI-I	CHOCI-R<br>COIS-R	CHOCI-R<br>COIS-R
**Comorbidity**	MINI-KID	SCAS<br>SDQ<br>CDI	SCAS<br>SDQ
**Global functioning**	CGAS		
**Parental psychopathology**			GAD-7<br>PHQ-9<br>OCI-R
**Family factors**	Family history of OCD / other diagnosis?		FAS-PR

1.2. Procedure

For recruitment, the project will be advertised in media, mental health services, primary care and schools. Inclusion procedures will be carried out in two steps: telephone interview and face-to-face assessment. Figure 1 gives an overview of the inclusion, assessment and treatment procedures.

1.2.1. Telephone interview

An initial telephone interview will be conducted with the parent/primary caregiver in order to assess broad inclusion and exclusion criteria. Participants will be interviewed with the MINI-KID screening items for OCD, which means asking the parent about the adolescents' obsessions and compulsions on a general level.

Inclusion criteria on this stage are:

- age between 12 and 17 years
- ability to read and write Swedish
- daily access to the internet
- a parent that is able to co-participate in the treatment
- Participants on psychotropic medication must have been on a stable dose for the last 6 weeks
- Suspected or likely compulsions or obsessions on the MINI-KID OCD screening

Subjects will be excluded on this stage if:

- subject not able to read or understand the basics of the ICBT self-help material
- psychotropic medication changes within 6 weeks prior to treatment
- completed CBT for OCD within last 12 months
- ongoing psychological treatment for OCD or another anxiety disorder
- Absence of compulsions and obsessions on the MINI-KID OCD screening
- presence of an autism spectrum disorder, psychosis/schizophrenia or bipolar disorder

1.2.2. Face-to-face baseline diagnostic assessment

After telephone screening participants are invited to a face-to-face assessment, including the diagnostic screening interview MINI-KID jointly with both the adolescent and parent. If MINI-KID indicates OCD, adolescent and parent will be interviewed jointly with CY-BOCS to assess OCD severity with respect to cut-off for inclusion (total score of > 15). Following face-to-face assessment, participants meeting inclusion criteria will be provided with an information sheet and consent form.

Inclusion criteria on this stage are:

- a primary diagnosis of OCD as defined by DSM-IV TR
- CY-BOCS total score > 15
- signed informed consent

Subjects will be excluded if:

- presence of an autism spectrum disorder, psychosis/schizophrenia or bipolar disorder
- suicidal ideation
- ongoing substance dependence
- CY-BOCS total score < 16

1.2.3. Baseline self-report measures

Participants included in the study after face-to-face diagnostic assessment will be asked to fill in self-report measures on the internet, provided via personal log-in.

1.2.4. Treatment

When completed the self-report measures adolescents and parents will be provided with login information for the ICBT treatment platform. Adolescents and parents will have separated login accounts. From the start of treatment participants will have full-scale access to the internet treatment for 12 weeks, which includes materials, interactive scripts and mail contact with a

clinician. After 12 weeks participants will have access to treatment materials, but it will not be possible to unlock new parts of the treatment or to have email contact with the clinician.

1.2.5. Weekly assessment of OC symptom severity

Participants will be assessed with the CHOCI-R parent and child version once a week to monitor treatment progress.

1.2.6. Safety procedures

Clinicians that suspect any kinds of adverse events under the assessment or treatment process contact the parent and adolescent via telephone as a first step of assessing the severity of the incident. Adverse events in this context is defined as an actual or potential situation that threaten the patients well-being e.g. suicide risk. In the case of an adverse event the clinician informs the study coordinator (FL) for discussion of adequate action taking. If prolonged ICBT treatment is considered inappropriate with regard to the participants' best interest and well-being the participant is excluded from the study and both parent and adolescent provided with proper referral information. Any adverse events will be reported in the planned publication of the pilot study.

1.2.7. Outcome measure reliability procedures

To ensure the reliability of the clinicians involved in MINI-KID and CY-BOCS interviews, clinicians will be trained by experienced instructors, independently rate and jointly discuss videotaped interviews. Under the assessment process, all baseline MINI-KID and CY-BOCS interviews will be taped and a random sample of 20% will be blindly scored by an independent evaluator. Inter-rater coefficients will be reported in the study article.

1.2.8. Post-treatment measurement and follow-up

Post-treatment measurement with clinician rated symptom severity (face-to-face interview with CY-BOCS, CGI-I, CGI-S and CGAS) as well as self-report measures will be conducted immediately after treatment, latest within 4 weeks after treatment protocol is finished.

Follow-up measurements will be performed at 3, 6 and 12 months after treatment. At follow-up CY-BOCS, CGI-I, CGI-S and CGAS will be administrated over the phone by a clinician. Self-report measures will be administrated via the internet.

Succeeding completed follow-up all participants will be contacted by the clinician via the ICBT platform and offered an ICBT booster session. Thus, standardized booster sessions will be offered at 3, 6 and 12 months after treatment.

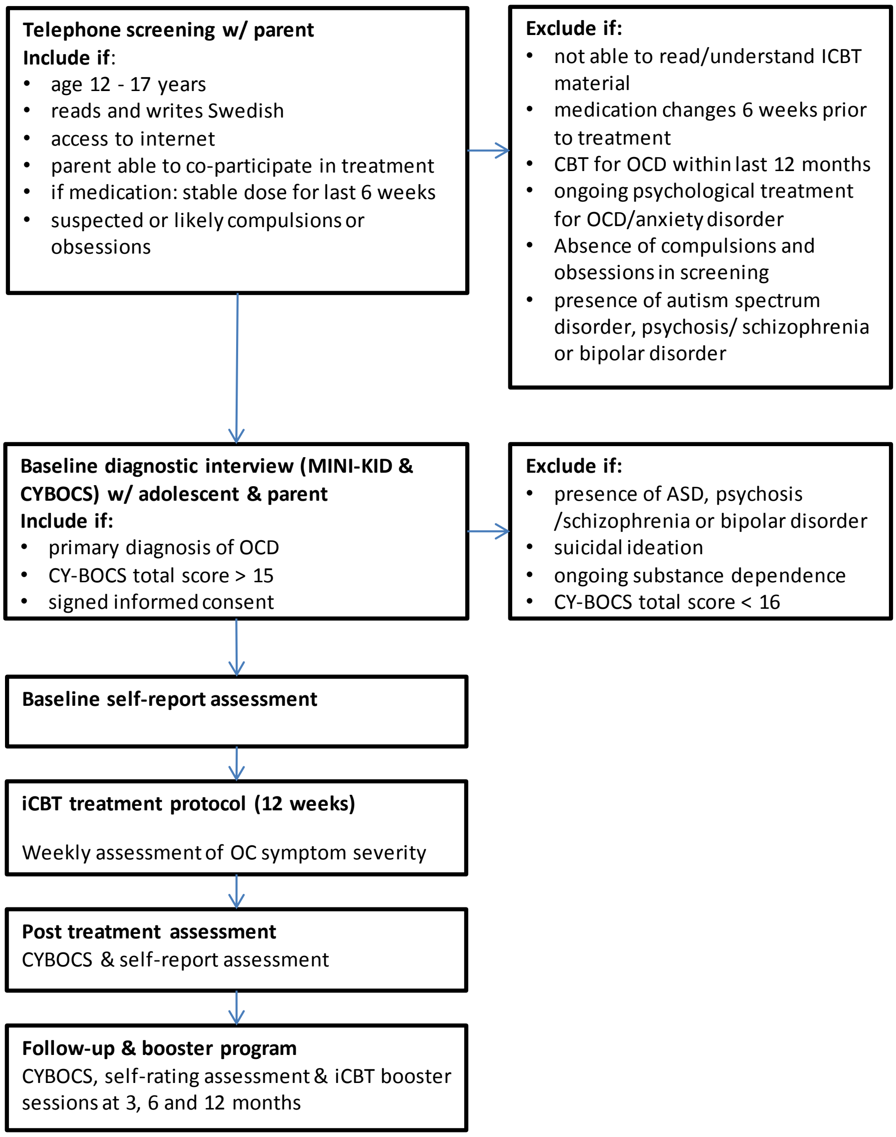

Figure 1: Procedures flow chart

1.3. ICBT treatment

The ICBT treatment program is completely web-based and will involve both the adolescent as well as the parents, consisting of evidence based interventions (Barrett, Healy-Farrell, & March, 2004; Bolton & Perrin, 2008; J. S. March, Mulle, & Herbel, 1994; Piacentini et al., 2011; The Pediatric OCD Treatment Study (POTS) Team, 2004; Williams et al., 2009). The program consists of texts to read, films and illustrations to watch as well as different kinds of exercises for the parents to do on their own and together with the adolescent. The program is divided into different steps, starting with psychoeducation regarding OCD and the rationale for cognitive behavioural interventions. Step two is the main part of treatment and contains behavioural interventions, mainly exposure with response prevention, as well as cognitive restructuring. In exposure with response prevention the patient confronts him-/herself with situations that normally trigger obsessions and learns how to prevent compulsions in order to habituate to anxiety. Cognitive restructuring is used to detect and modify

dysfunctional and anxiety evoking thinking patterns and replace them with more rational thoughts. Step three addresses problem solving, maintenance of treatment gains and relapse prevention. Parents and adolescents will basically follow the same structure in their respective parts of treatment. In addition to the above named steps will the parent protocol focus on family accommodation, parental coping strategies and how to coach the adolescent through treatment. Parents and adolescents will have separate login accounts. The adolescent's self-help manual is especially age-designed with interactive scripts. During the treatment period, which is twelve weeks, participants will have regular contact with the therapist through e-mails, phone calls and standardized forms in the program. One intention when developing the program is to make it as easily accessible as possible so that not only Swedish families but also children and parents who don't have Swedish as their mother tongue will be able to benefit from it.

1.4. Statistical analysis

In a first treatment step, we will test the feasibility of a 12-week ICBT for OCD in a pilot study using an open trial design (n=27, d=0.5, 80% power, p=.05).

1.5. Planned publications

Results are planned to be published as an article in a scientific journal: Internet-delivered CBT for adolescents with obsessive-compulsive disorder: a pilot study.

1.6. Project schedule

2012 Q3 - Q4: Development of ICBT protocol and technical platform, planning for pilot study, ethical application, training of therapists.

2013 Q1 - Q2: Pilot study of protocol, platform and research procedures.

2013 Q3 – 2014 Q2: Evaluation of pilot study and follow up.

2. Anticipated effects

The overall goal of our research is to develop a new format of effective treatment for paediatric OCD. This pilot study contributes with information about feasibility and preliminary effectiveness of the ICBT treatment. In the long run we believe that ICBT could have the potential to increase the availability of treatment of OCD in adolescents both in a geographical and temporal sense. This would be desirable not only for patients and their families, but also for health care establishments, who could decrease their waiting times. Moreover, internet-delivered interventions potentially supply effective treatment at a lower cost.

3. Ethical considerations

ICBT differs from traditional therapy in the amount of information that the therapist is given. A thorough assessment decreases the risk of patients in need of more extensive care being included in the study. The regular e-mail communication, and the possibility for the participating families to contact their therapist if needed, ensures that families with problems to severe for this program will be referred to more suitable treatments. The participants may worry about computer safety and maintenance of confidentiality. The families are provided with information about the risks and precautions that are being taken when using communication technology, e.g. encrypted server technology. International rules and regulations will be followed. In our view, the ethical risk is limited since every study participant will receive treatment.

4. Presentation of research group

The members of the research group have extensive experience from clinical work with children with anxiety and OCD disorders as well as research and clinical implementation of internet-delivered CBT treatment. All investigators have at least 50% planned research time. **Eva Serlachius**, Ph.D. M.D., is a child psychiatrist and principal investigator. She is as well responsible for integration of the project into the child and adolescent psychiatry clinic. **Christian Rück,** Ph.D. M.D**,** psychiatrist at the OCD unit for adults at Psykiatri Sydväst and has recently published the first clinical trial on ICBT for adults with OCD. CR provides expertise in clinical trial design for OCD and execution. **David Mataix-Cols,** Professor at the Institute of Psychiatry, King's College London, and Maudsley Hospital, accomplished OCD and related disorders researcher with extensive trial experience in children and adolescents. **Ulrika Thulin,** PhD, licensed psychologist and psychotherapist with extensive CBT training and previous experience of development of ICBT manuals. **Sarah Vigerland**, PhD student, licensed psychologist has previous experience from developing internet-administrated treatment for insomnia and is coordinator for the development of ICBT for children with anxiety disorders. **Erik Andersson**, licensed psychologist, PhD student, working at the OCD unit for adults at Psykiatri sydväst, currently conducting research on ICBT for adults with OCD. **Fabian Lenhard**, licensed psychologist, psychotherapist (CBT) and PhD student has long experience of treating children and adolescents with OCD and is project manager for the pilot study.

4. References

Abramowitz, J. S., Whiteside, S. P., & Deacon, B. J. (2000). The Effectiveness of Treatment for Pediatric Obsessive-Compulsive Disorder : A Meta-Analysis.

American Psychiatric Association. (1994). *Diagnostic and Statistical Manual of Mental Disorders (4th edn) (DSM–IV)*. APA.

Andersson, E., Enander, J., Andrén, P., Hedman, E., Ljótsson, B., Hursti, T., Bergström, J., et al. (2012). Internet-based cognitive behaviour therapy for obsessive-compulsive disorder: a randomized controlled trial. *Psychological medicine*, 1–11. doi:10.1017/S0033291712000244

Andersson, G. (2009). Using the Internet to provide cognitive behaviour therapy. *Behaviour research and therapy*, *47*(3), 175–80. doi:10.1016/j.brat.2009.01.010

Andersson, G., Bergström, J., Holländare, F., Carlbring, P., Kaldo, V., & Ekselius, L. (2005). Internet-based self-help for depression: randomised controlled trial. *The British journal of psychiatry*, *187*, 456–61. doi:10.1192/bjp.187.5.456

Angst, J., Gamma, A., Endrass, J., Goodwin, R., Ajdacic, V., Eich, D., & Rössler, W. (2004). Obsessive-compulsive severity spectrum in the community: prevalence, comorbidity, and course. *European archives of psychiatry and clinical neuroscience*, *254*(3), 156–64. doi:10.1007/s00406-004-0459-4

Barrett, P., Healy-Farrell, L., & March, J. S. (2004). Cognitive-behavioral family treatment of childhood obsessive-compulsive disorder: a controlled trial. *Journal of the American Academy of Child and Adolescent Psychiatry*, *43*(1), 46–62. doi:10.1097/00004583-200401000-00014

Bergström, J., Andersson, G., Ljótsson, B., Rück, C., Andréewitch, S., Karlsson, A., Carlbring, P., et al. (2010). Internet-versus group-administered cognitive behaviour therapy for panic disorder in a psychiatric setting: a randomised trial. *BMC psychiatry*, *10*, 54. doi:10.1186/1471-244X-10-54

Bolton, D., & Perrin, S. (2008). Evaluation of exposure with response-prevention for obsessive compulsive disorder in childhood and adolescence. *Journal of behavior therapy and experimental psychiatry*, *39*(1), 11–22. doi:10.1016/j.jbtep.2006.11.002

Calvocoressi, L., Mazure, C. M., Kasl, S. V., Skolnick, J., Fisk, D., Vegso, S. J., Van Noppen, B. L., et al. (1999, October). Ovid: Family Accommodation of Obsessive-Compulsive Symptoms: Instrument Development and Assessment of Family Behavior. *The Journal of nervous and mental disease*. Retrieved February 9, 2012, from http://ovidsp.uk.ovid.com/sp-3.5.1a/ovidweb.cgi?&S=GDFNPDDGFNHFMMJPFNALJFOFKKGHAA00&Link+Set=S.sh.15|1|sl_11129619

Flament, M. F., Whitaker, A., Rapoport, J. L., Davies, M., Berg, C. Z., Kalikow, K., Sceery, W., et al. (1988). Obsessive compulsive disorder in adolescence: an epidemiological study. *Journal of the American Academy of Child and Adolescent Psychiatry*, *27*(6), 764–71. doi:10.1097/00004583-198811000-00018

Flessner, C. A., Sapyta, J., Garcia, A., Freeman, J. B., Franklin, M. E., Foa, E., & March, J. (2009). Examining the Psychometric Properties of the Family Accommodation Scale-Parent-Report (FAS-PR). *Journal of psychopathology and behavioral assessment*, *31*(1), 38–46.

Foa, E. B., Huppert, J. D., Leiberg, S., Langner, R., Kichic, R., Hajcak, G., & Salkovskis, P. M. (2002). The Obsessive-Compulsive Inventory: development and validation of a short version. *Psychological assessment*, *14*(4), 485–96. Retrieved from http://www.ncbi.nlm.nih.gov/pubmed/12501574

Garcia, A. M., Sapyta, J. J., Moore, P. S., Freeman, J. B., Franklin, M. E., March, J. S., & Foa, E. B. (2010). Predictors and moderators of treatment outcome in the Pediatric Obsessive Compulsive Treatment Study (POTS I). *Journal of the American Academy of Child and Adolescent Psychiatry*, *49*(10), 1024–33; quiz 1086. doi:10.1016/j.jaac.2010.06.013

Geller, D. A., Biederman, J., Stewart, S. E., Mullin, B., Martin, A., Spencer, T., & Faraone, S. V. (2003). Which SSRI? A meta-analysis of pharmacotherapy trials in pediatric obsessive-compulsive disorder. *The American journal of psychiatry*, *160*(11), 1919–28. Retrieved from http://www.ncbi.nlm.nih.gov/pubmed/14594734

Ginsburg, G. S., Kingery, J. N., Drake, K. L., & Grados, M. A. (2008). Predictors of treatment response in pediatric obsessive-compulsive disorder. *Journal of the American Academy of Child and Adolescent Psychiatry*, *47*(8), 868–78. doi:10.1097/CHI.0b013e3181799ebd

Goodman, R. (1997). The Strengths and Difficulties Questionnaire: a research note. *Journal of child psychology and psychiatry, and allied disciplines*, *38*(5), 581–6. Retrieved from http://www.ncbi.nlm.nih.gov/pubmed/9255702

Goodman, R., Ford, T., Richards, H., Gatward, R., & Meltzer, H. (2000). The Development and Well-Being Assessment: description and initial validation of an integrated assessment of child and adolescent psychopathology. *Journal of child psychology and psychiatry, and allied disciplines*, *41*(5), 645–55. Retrieved from http://www.ncbi.nlm.nih.gov/pubmed/10946756

Goodwin, R., Koenen, K. C., Hellman, F., Guardino, M., & Struening, E. (2002). Helpseeking and access to mental health treatment for obsessive-compulsive disorder. *Acta psychiatrica Scandinavica*, *106*(2), 143–9. Retrieved from http://www.ncbi.nlm.nih.gov/pubmed/12121213

Guy, W. (1976). *Assessment manual for psychopharmacology, revised*. Washington DC: US Government Printing Office.

Hedman, E., Andersson, G., Andersson, E., Ljótsson, B., Rück, C., Asmundson, G. J. G., & Lindefors, N. (2011). Internet-based cognitive-behavioural therapy for severe health anxiety: randomised controlled trial. *The British journal of psychiatry : the journal of mental science*, *198*(3), 230–6. doi:10.1192/bjp.bp.110.086843

Hedman, E., Andersson, G., Ljótsson, B., Andersson, E., Rück, C., Mörtberg, E., & Lindefors, N. (2011). Internet-based cognitive behavior therapy vs. cognitive behavioral group therapy for social anxiety disorder: a randomized controlled non-inferiority trial. *PloS one*, *6*(3), e18001. doi:10.1371/journal.pone.0018001

Kovacs, M. (1985). The Children's Depression, Inventory (CDI). *Psychopharmacology bulletin*, *21*(4), 995–8. Retrieved from http://www.ncbi.nlm.nih.gov/pubmed/4089116

Kroenke, K., Spitzer, R. L., & Williams, J. B. (2001). The PHQ-9: validity of a brief depression severity measure. *Journal of general internal medicine*, *16*(9), 606–13. Retrieved from http://www.pubmedcentral.nih.gov/articlerender.fcgi?artid=1495268&tool=pmcentrez&rendertype=abstract

Lundh, A., Kowalski, J., Sundberg, C. J., Gumpert, C., & Landén, M. (2010). Children's Global Assessment Scale (CGAS) in a naturalistic clinical setting: Inter-rater reliability and comparison with expert ratings. *Psychiatry research*, *177*(1-2), 206–10. doi:10.1016/j.psychres.2010.02.006

March, J. S., Mulle, K., & Herbel, B. (1994). Behavioral psychotherapy for children and adolescents with obsessive-compulsive disorder: an open trial of a new protocol-driven treatment package. *Journal of the American Academy of Child and Adolescent Psychiatry*, *33*(3), 333–41. doi:10.1097/00004583-199403000-00006

March, S., Spence, S. H., & Donovan, C. L. (2009). The efficacy of an internet-based cognitive-behavioral therapy intervention for child anxiety disorders. *Journal of pediatric psychology*, *34*(5), 474–87. doi:10.1093/jpepsy/jsn099

Micali, N., Heyman, I., Perez, M., Hilton, K., Nakatani, E., Turner, C., & Mataix-Cols, D. (2010). Long-term outcomes of obsessive-compulsive disorder: follow-up of 142 children and adolescents. *The British journal of psychiatry : the journal of mental science*, *197*(2), 128–34. doi:10.1192/bjp.bp.109.075317

National Institute of Mental Health. (1985). Rating scales and assessment instruments for use in pediatric psychopharmacology research. *Psychopharmacol Bull*, *21*, 839–843.

Piacentini, J., Bergman, R. L., Chang, S., Langley, A., Peris, T., Wood, J. J., & McCracken, J. (2011). Controlled comparison of family cognitive behavioral therapy and psychoeducation/relaxation training for child obsessive-

compulsive disorder. *Journal of the American Academy of Child and Adolescent Psychiatry*, *50*(11), 1149–61. doi:10.1016/j.jaac.2011.08.003

Piacentini, J., Peris, T. S., Bergman, R. L., Chang, S., & Jaffer, M. (2007). Functional impairment in childhood OCD: development and psychometrics properties of the Child Obsessive-Compulsive Impact Scale-Revised (COIS-R). *Journal of clinical child and adolescent psychology : the official journal for the Society of Clinical Child and Adolescent Psychology, American Psychological Association, Division 53*, *36*(4), 645–53. doi:10.1080/15374410701662790

Pinto, A., Mancebo, M. C., Eisen, J. L., Pagano, M. E., & Rasmussen, S. A. (2006). The Brown Longitudinal Obsessive Compulsive Study: clinical features and symptoms of the sample at intake. *The Journal of clinical psychiatry*, *67*(5), 703–11. Retrieved from http://www.pubmedcentral.nih.gov/articlerender.fcgi?artid=3272757&tool=pmcentrez&rendertype=abstract

Reynolds, S., Wilson, C., Austin, J., & Hooper, L. (2012). Clinical Psychology Review Effects of psychotherapy for anxiety in children and adolescents : A meta-analytic review. *Clinical Psychology Review*, *32*(4), 251–262. doi:10.1016/j.cpr.2012.01.005

Scahill, L., Riddle, M. A., McSwiggin-Hardin, M., Ort, S. I., King, R. A., Goodman, W. K., Cicchetti, D., et al. (1997). Children's Yale-Brown Obsessive Compulsive Scale: reliability and validity. *Journal of the American Academy of Child and Adolescent Psychiatry*, *36*(6), 844–52. doi:10.1097/00004583-199706000-00023

Schneider, A. J., Mataix-Cols, D., Marks, I. M., & Bachofen, M. (2005). Internet-guided self-help with or without exposure therapy for phobic and panic disorders. *Psychotherapy and psychosomatics*, *74*(3), 154–64. doi:10.1159/000084000

Shaffer, D., Gould, M. S., Brasic, J., Ambrosini, P., Fisher, P., Bird, H., & Aluwahlia, S. (1983). A children's global assessment scale (CGAS). *Archives of general psychiatry*, *40*(11), 1228–31. Retrieved from http://www.ncbi.nlm.nih.gov/pubmed/6639293

Shafran, R., Frampton, I., Heyman, I., Reynolds, M., Teachman, B., & Rachman, S. (2003). The preliminary development of a new self-report measure for OCD in young people. *Journal of adolescence*, *26*(1), 137–42. Retrieved from http://www.ncbi.nlm.nih.gov/pubmed/12550826

Sheehan, D V, Lecrubier, Y., Sheehan, K. H., Amorim, P., Janavs, J., Weiller, E., Hergueta, T., et al. (1998). The Mini-International Neuropsychiatric Interview (M.I.N.I.): the development and validation of a structured diagnostic psychiatric interview for DSM-IV and ICD-10. *The Journal of clinical psychiatry*, *59 Suppl 2*, 22–33;quiz 34–57. Retrieved from http://www.ncbi.nlm.nih.gov/pubmed/9881538

Sheehan, David V, Sheehan, K. H., Shytle, R. D., Janavs, J., Bannon, Y., Rogers, J. E., Milo, K. M., et al. (2010). Reliability and validity of the Mini International Neuropsychiatric Interview for Children and Adolescents (MINI-KID). *The Journal of clinical psychiatry*, *71*(3), 313–26. doi:10.4088/JCP.09m05305whi

Socialstyrelsen. (2009). *Nationella riktlinjer för depressionssjukdom och ångestsyndrom – beslutsstöd för prioriteringar, Preliminär version*. SoS, Editor.

Spek, V., Cuijpers, P., Nyklícek, I., Riper, H., Keyzer, J., & Pop, V. (2007). Internet-based cognitive behaviour therapy for symptoms of depression and anxiety: a meta-analysis. *Psychological medicine*, *37*(3), 319–28. doi:10.1017/S0033291706008944

Spence, S. H. (1998). A measure of anxiety symptoms among children. *Behaviour research and therapy*, *36*(5), 545–66. Retrieved from http://www.ncbi.nlm.nih.gov/pubmed/9648330

Spitzer, R. L., Kroenke, K., Williams, J. B. W., & Löwe, B. (2006). A brief measure for assessing generalized anxiety disorder: the GAD-7. *Archives of internal medicine*, *166*(10), 1092–7. doi:10.1001/archinte.166.10.1092

Storch, E. A., Murphy, T. K., Geffken, G. R., Soto, O., Sajid, M., Allen, P., Roberti, J. W., et al. (2004). Psychometric evaluation of the Children's Yale-Brown Obsessive-Compulsive Scale. *Psychiatry research*, *129*(1), 91–8. doi:10.1016/j.psychres.2004.06.009

The Pediatric OCD Treatment Study (POTS) Team. (2004). Cognitive-behavior therapy, sertraline, and their combination for children and adolescents with obsessive-compulsive disorder: the Pediatric OCD Treatment Study (POTS) randomized controlled trial. *JAMA : the journal of the American Medical Association*, *292*(16), 1969–76. doi:10.1001/jama.292.16.1969

Uher, R., Heyman, I., Turner, C. M., & Shafran, R. (2008). Self-, parent-report and interview measures of obsessive-compulsive disorder in children and adolescents. *Journal of anxiety disorders*, *22*(6), 979–90. doi:10.1016/j.janxdis.2007.10.001

Valderhaug, R., Götestam, K., & Larsson, B. (2004). Clinicians' views on management of obsessive-compulsive disorders in children and adolescents. *Nordic journal of psychiatry*, *58*(2), 125–32. doi:10.1080/08039480410005503

Valderhaug, R., & Ivarsson, T. (2005). Functional impairment in clinical samples of Norwegian and Swedish children and adolescents with obsessive-compulsive disorder. *European child & adolescent psychiatry*, *14*(3), 164–73. doi:10.1007/s00787-005-0456-9

Valleni-Basile, L. A., Garrison, C. Z., Jackson, K. L., Waller, J. L., McKeown, R. E., Addy, C. L., & Cuffe, S. P. (1994). Frequency of obsessive-compulsive disorder in a community sample of young adolescents. *Journal of the American Academy of Child and Adolescent Psychiatry*, *33*(6), 782–91. doi:10.1097/00004583-199407000-00002

Wahl, K., Kordon, A., Kuelz, K. A., Voderholzer, U., Hohagen, F., & Zurowski, B. (2010). Obsessive-Compulsive Disorder (OCD) is still an unrecognised disorder: a study on the recognition of OCD in psychiatric outpatients. *European psychiatry : the journal of the Association of European Psychiatrists*, *25*(7), 374–7. doi:10.1016/j.eurpsy.2009.12.003

Watson, H. J., & Rees, C. S. (2008). Meta-analysis of randomized, controlled treatment trials for pediatric obsessive-compulsive disorder. *Journal of child psychology and psychiatry, and allied disciplines*, *49*(5), 489–98. doi:10.1111/j.1469-7610.2007.01875.x

Wewetzer, C., Jans, T., Müller, B., Neudörfl, A., Bücherl, U., Remschmidt, H., Warnke, A., et al. (2001). Long-term outcome and prognosis of obsessive-compulsive disorder with onset in childhood or adolescence. *European child & adolescent psychiatry*, *10*(1), 37–46. Retrieved from http://www.ncbi.nlm.nih.gov/pubmed/11315534

Williams, T. I., Salkovskis, P. M., Forrester, L., Turner, S., White, H., & Allsopp, M. a. (2009). A randomised controlled trial of cognitive behavioural treatment for obsessive compulsive disorder in children and adolescents. *European Child & Adolescent Psychiatry*, *19*(5), 449–456. doi:10.1007/s00787-009-0077-9
